# *Candida* species-specific colonization in the healthy and impaired human gastrointestinal tract as simulated using the Mucosal Ileum-SHIME^®^ model

**DOI:** 10.1093/femsec/fiae113

**Published:** 2024-08-21

**Authors:** Benoît Marsaux, Frédéric Moens, Gies Vandevijver, Massimo Marzorati, Tom van de Wiele

**Affiliations:** ProDigest B.V., 9052 Ghent, Belgium; CMET, Ghent University, 9000 Ghent, Belgium; ProDigest B.V., 9052 Ghent, Belgium; ProDigest B.V., 9052 Ghent, Belgium; ProDigest B.V., 9052 Ghent, Belgium; CMET, Ghent University, 9000 Ghent, Belgium; ProDigest B.V., 9052 Ghent, Belgium; CMET, Ghent University, 9000 Ghent, Belgium

**Keywords:** antibiotic, *Candida* species, commensal, dysbiosis, *in vitro* models, interkingdom interactions, pathogenic

## Abstract

*Candida* species primarily exist as harmless commensals in the gastrointestinal tract of warm-blooded animals. However, they can also cause life-threatening infections, which are often associated with gut microbial dysbiosis. Identifying the microbial actors that restrict *Candida* to commensalism remains a significant challenge. *In vitro* models could enable a mechanistic study of the interactions between *Candida* and simulated colon microbiomes. Therefore, this study aimed to elucidate the spatial and temporal colonization kinetics of specific *Candida*, including *C. albicans, C. tropicalis*, and *C. parapsilosis*, and their relative *Nakaseomyces glabratus*, by using an adapted SHIME® model, simulating the ileum, and proximal and distal colons. We monitored fungal and bacterial colonization kinetics under conditions of eubiosis (commensal lifestyle) and antibiotic-induced dysbiosis (pathogenic lifestyle). Our findings highlighted the variability in the colonization potential of *Candida* species across different intestinal regions. The ileum compartment proved to be the most favourable environment for *C. albicans* and *C. parapsilosis* under conditions of eubiosis. Antibiotic-induced dysbiosis resulted in resurgence of opportunistic *Candida* species, especially *C. tropicalis* and *C. albicans*. Future research should focus on identifying specific bacterial species influencing *Candida* colonization resistance and explore the long-term effects of antibiotics on the mycobiome and bacteriome.

## Teaser

The multicompartmental semidynamic SHIME® model was used to elucidate the spatial and temporal colonization kinetics of specific *Candida* species under conditions of eubiosis and dysbiosis.

## Introduction

Between 2.2 and 3.8 million fungal species inhabit Earth, colonizing a diverse range of ecological niches, including various sites both on and within the human body (Underhill and Iliev 2014, Hawksworth and Lucking 2017). Among these fungi, several strains can pose significant health risks to humans, resulting in a global health burden (Bongomin et al. 2017). Approximately 25% of the world’s population suffers from skin infections, and about 75% of women will experience at least one episode of vulvovaginal candidiasis during their lifetime (Brown et al. 2012). Furthermore, fungal systemic infections contribute to mortality rates ranging from 10% to 47% (Brown et al. 2012). Recognizing this, the World Health Organization (WHO) recently issued its first list of top-priority fungal pathogens, with *Candida* species prominently featured (Brown et al. 2012). Notably, *Candida albicans*, responsible for 80% of human candidiasis cases (Silva et al. 2012), is among the WHO’s four critical priority fungal species, while *Candida tropicalis, Candida parapsilosis*, and *Nakaseomyces glabratus* (formerly *Candida glabrata*; Takashima and Sugita 2022, which we considered a *Candida* for ease of writing) are listed within the seven high-priority ones.

*Candida* species primarily exist as harmless commensals in the gastrointestinal tract of warm-blooded animals, particularly birds and mammals (Odds 1984). However, the *Candida* genus can also comprise pathogens and pathobionts, which can cause life-threatening infections. These infections mainly affect immunocompromised individuals and are often associated with microbial dysbiosis (Koh et al. 2008, Zhai et al. 2020, Li et al. 2022). Hence, both the host immune system and the gut microbiome play strategic roles in preventing the commensal-to-pathogen transition in *Candida* species (d’Enfert et al. 2021). In particular, the gut microbiome has a pivotal role in preventing candidiasis, serving as the first line of defense against pathological *Candida* outgrowth through a phenomenon termed “colonization resistance” (Lawley and Walker 2013). This colonization resistance involves microbe–microbe interactions, such as competition for nutrients, niches, and binding sites, as well as the release of antimicrobial agents (Dabard et al. 2001, Momose et al. 2008, Gong et al. 2010, Rea et al. 2010, Deriu et al. 2013, Wagner 2022, Xu et al. 2023). However, identifying the key microbial actors that restrict *Candida* to commensalism remains a significant challenge. The same can be said for comprehending the impact of antibiotic-associated dysbiosis in candidiasis pathology.

Current models available to study *Candida*–gut bacteriome interactions have drawbacks. On the one hand, mouse models, historically reliant on antibiotic treatment to establish stable *Candida* colonization in the gut (Fan et al. 2015), are limited in accurately replicating *Candida*’s commensal behaviour since the microbiome should be impaired to obtain *C. albicans* colonization (Neville et al. 2015, Perez 2019). On the other hand, *in vitro* models used to study *Candida*–bacteria interactions in bioreactors face challenges in cultivating faecal-derived strict anaerobes alongside oxygen-dependent fungi (Auchtung et al. 2018). Addressing this issue may require separate cultivation of strict anaerobes and *Candida* (Ricci et al. 2022), which precludes investigating direct interkingdom interactions, lowering its relevance.

Promisingly, Payne and colleagues (Payne et al. 2003, Wynne et al. 2004) studied *C. albicans* within a faecal-derived microbiome background using a multistage fermentation model two decades ago, yet no follow-up studies ensued. To date, only Maas and colleagues, and our research group, have explored bacteria–fungi interactions in faecal-derived microbiomes, using the TIM-2 and the SHIME® models (with features reviewed in Van de Wiele et al. 2015), respectively (Maas et al. 2023a, b, Marsaux et al. 2023). In particular, we explored the natural engraftment of fungi in both eubiosis (commensal lifestyle) and dysbiosis (pathogenic lifestyle) states, and we highlighted the critical role of certain bacterial species in preventing fungal outgrowth (Marsaux et al. 2023). However, despite examining numerous faecal samples, only one individual contained *Candida* species, which was eventually washed out from the system under eubiosis conditions. Similarly, *C. albicans* did not consistently grow in the TIM-2 model (Maas et al. 2023a, b). Hence, there is a pressing need to design complex *in vitro* models, which do not rely on the presence of *Candida* in the faecal inocula that will enable a mechanistic study of the interactions between *Candida* and simulated colon microbiomes.

In the present study, we used an adapted SHIME® model to explore whether any *Candida* species of interest can be studied alongside individual colonic microbiomes, derived from a human faecal sample. Specifically, we sought to decipher the ecological factors influencing *Candida* colonization by artificially inoculating the SHIME® model with species that have been linked to pathogenic properties, including *C. albicans, C. tropicalis*, and *C. parapsilosis*, as well as their relative *N. glabratus*. As a control to distinguish active colonization from transient passage upon artificial inoculation, we also inoculated *Saccharomyces cerevisiae*, a transient microbe of the gut microbiome (Auchtung et al. 2018). To investigate the longitudinal colonization ability of the different *Candida* species along the simulated human gastrointestinal tract, we used the recently developed Mucosal Ileum-SHIME® model (Deyaert et al. 2022). This model allowed us to assess colonization dynamics in the simulated ileum, proximal colon, and distal colon, all in the presence of a mucosal compartment, which has been shown to be essential for maintaining crucial functional niches and the unique features of an individual’s gut microbiome (Van den Abbeele et al. 2013). We monitored fungal and bacterial colonization kinetics in both eubiosis (commensal lifestyle) and upon antibiotic-induced disruption (pathogenic lifestyle). 16S rRNA gene-targeted Illumina sequencing was conducted to evaluate the bidirectional impact of *Candida* engraftment and antibiotic administrations on bacteriome diversity. Finally, to account for substantial interindividual variability across the population, we initiated the experiment starting from distinct faecal inocula obtained from three healthy individuals. Overall, this study serves as a proof of concept for using the Mucosal Ileum-SHIME® model to investigate *Candida*–bacteria interactions and sets the stage for discovering factors contributing to *Candida* outgrowth and commensal-to-pathogen transition.

## Materials and methods

### Chemical products

All chemicals were obtained from Merck (Darmstadt, Germany) unless otherwise stated.

### The *in vitro* Mucosal Ileum-SHIME^®^ technology

**Figure 1.**
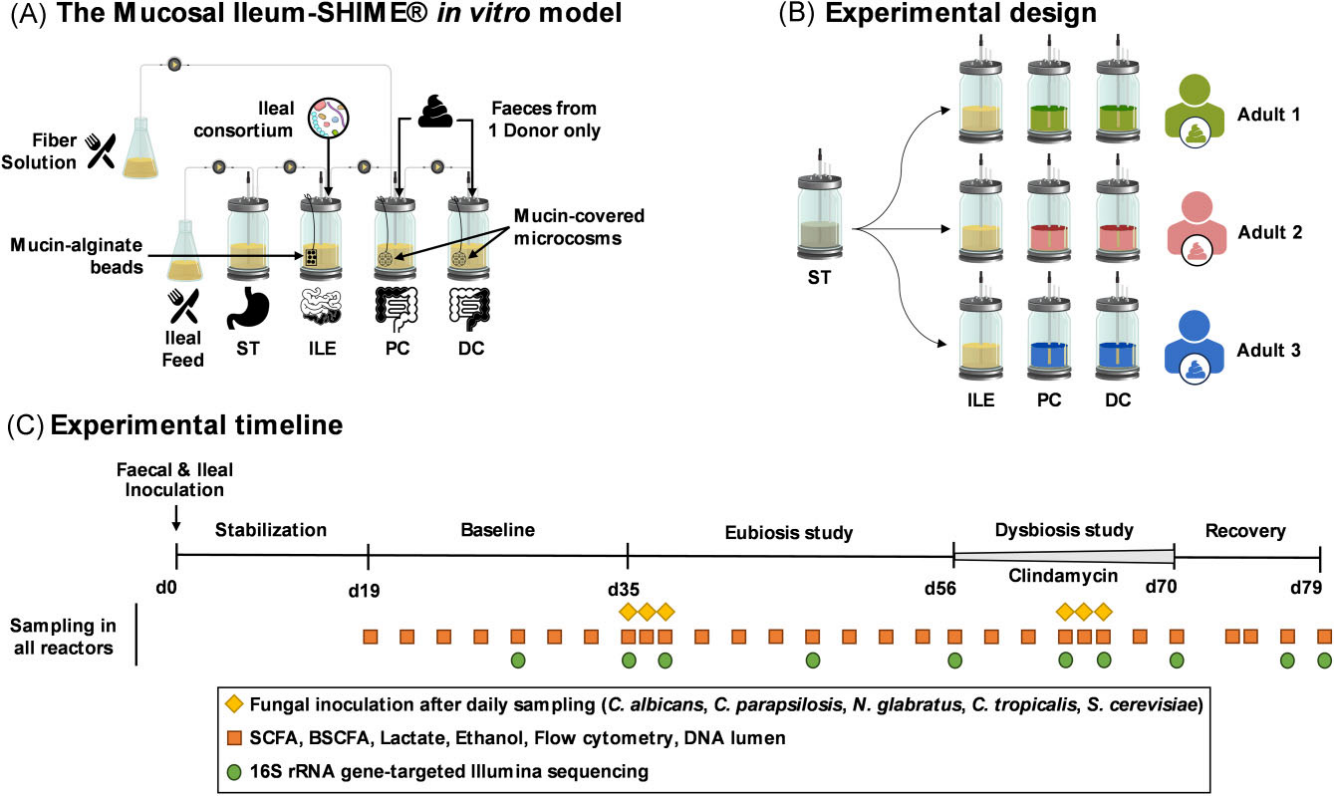
Schematic representation of the Mucosal Ileum-SHIME® *in vitro* model (A), the experimental design (B), and the experimental timeline (C). ST = stomach; ILE = ileum; PC = proximal colon; DC = distal colon; SHIME® = Simulator of the Human Intestinal Microbial Ecosystem; d = day; SCFA = short-chain fatty acid; and BSCFA = branched short-chain fatty acid.

The experiments were performed through modification of the M-SHIME® technology (ProDigest and Ghent University, Ghent, Belgium) previously described by Van den Abbeele et al. (2012). The proximal colon compartment was preceded with an ileum compartment in a similar approach as was recently published by our research group (Deyaert et al. 2022). The configuration of the Mucosal Ileum-SHIME® model is summarized in Fig. 1(A).

Briefly, the entire setup was autoclaved at 121°C for 20 min before inoculation to ensure sterility. Reactors were continuously stirred and maintained at 37°C. Anaerobic conditions were ensured by sparging the ileal nutritional medium with nitrogen (N_2_) for 15 min each time it was introduced into the stomach compartment three times a day. Additionally, the headspace of each reactor was flushed with N_2_ daily. The proximal colon compartments received 150 ml of ileal content three times a day after 1.5 h of fermentation in the ileum compartment, along with 50 ml of fiber solution. To simulate the Mucosal component of the human gastrointestinal tract, we introduced mucin-alginate beads in the ileum compartments and mucin-covered microcosms in the proximal and distal colon compartments. The preparation and replacement of these Mucosal components were performed as previously described (Deyaert et al. 2022). In short, mucin-alginate beads, which can be sampled from the ileum compartments in a sterile manner, were prepared by dripping a mucin-alginate solution into a cross-linking solution containing CaCl_2_ (Deyaert et al. 2022). In contrast, mucin-covered microcosms (length = 7 mm, diameter = 9 mm, total surface area = 800 m^2^ m^−3^, AnoxKaldnes K1 carrier, AnoxKaldnes AB, Lund, Sweden) were coated by submerging them in a mucin–agar solution (Van den Abbeele et al. 2012). Finally, the ileum compartments were inoculated at day 0 with a consortium of 12 bacterial species comprising *Streptococcus bovis, Streptococcus intermedius, Ligilactobacillus salivarius, Limosilactobacillus reuteri, Enterococcus faecalis, Enterococcus faecium, Veillonella parvula, Veillonella dispar, Blautia obeum, Faecalibacterium prausnitzii, Clostridium nexile, and Prevotella melaninogica* (Deyaert et al. 2022). They were selected to simulate the function and composition of a healthy individual’s ileal microbiome, including microbes performing partial conversion and deconjugation of bile salts, as well as primary fermenters metabolizing simple carbohydrates into e.g. lactate, that support the growth of secondary fermenters, producing e.g. short-chain fatty acid (SCFA) through cross-feeding (Deyaert et al. 2022). In contrast, the proximal and distal colon compartments were inoculated with the faecal sample of a healthy donor. Details regarding the growth conditions of the ileal bacterial consortium, as well as the preparation of the faecal inoculum, have been previously described (Deyaert et al. 2022).

The ileal nutritional medium was prepared by sterile addition (5% v/v) of a filter-sterilized stock solution containing (l^−1^) NaHCO_3_ (50 g; Chem-Lab NV, Zedelgem, Belgium), NaH_2_PO_4_ (10 g; Avantor™, PA, USA), K_2_HPO_4_ (10 g; Chem-Lab), MgSO_4_.7H_2_O (0.9 g; Chem-Lab), MnCl_2_.4H_2_O (0.5 g; Avantor™), CaCl_2_.2H_2_O (0.9 g; Avantor™), FeSO_4_.7H_2_O (0.05 g), ZnSO_4_.7H_2_O (0.05 g), hemin (0.05 g), glucose (4 g), fructose (4 g), sucrose (4 g), maltose (4 g; Carl Roth, Karlsruhe, Germany), lactose (4 g), mannose (2.5 g; Carl Roth), and galactose (2.5 g) to an autoclaved medium comprising (l^−1^) bile salts (1.8 g; Oxgall; BD Bioscience, Erembodegem, Belgium), special peptone (0.23 g), yeast extract (0.7 g), mucin (0.47 g; Carl Roth), l-cysteine-HCl (0.12 g; AXO Industry SA, Wavre, Belgium), and Tween®80 (1 ml). The fiber solution contained (g l^−1^) arabinogalactan (3.4; Keyser & Mackay, Amsterdam, The Netherlands), pectin (5.6; Keyser & Mackay), xylan (1.4; Carl Roth), starch (11.2; Carl Roth), glucose (1.12), special peptone (2.1), yeast extract (6.3), mucin (4.2; Carl Roth), and l-cysteine-HCl (1.0).

### Experimental design

#### Eubiosis study to elucidate the ability of five fungal species to colonize a healthy microbial community

The eubiosis study aimed to assess the colonization ability of five distinct fungal species within the gut microbiome under healthy conditions. The four *Candida* species, namely *C. albicans, C. tropicalis, N. glabratus*, and *C. parapsilosis*, were selected as they are among the most life-threatening fungi. As a control, *S. cerevisiae* was selected as model organism of transient passage, as it does not naturally colonize the gut microbiome *in vivo*. Consequently, *S. cerevisiae* can be used to differentiate active from transient passage of the four *Candida* species and to confirm that active colonization is not the result of artificial inoculation. Additionally, the eubiosis study sought to evaluate the impact of the five fungal species inoculation on the microbiome’s function and composition.

Briefly, the Mucosal Ileum-SHIME® reactors were initially inoculated either with an ileal bacterial consortium (ileum compartment) or the faecal sample from a single donor (proximal and distal colon compartments). The system received three feeding cycles each day. The study began with a stabilization period (d0–d19), during which the microbial community in each colon compartment diversified and eventually established stability. Following the stabilization period, a baseline period (d19–d35) allowed to study the baseline microbiota composition and metabolic activity before initiating any intervention. Subsequently, 1 ml of an overnight culture of each fungal species was inoculated once daily for three consecutive days (d35–d37) in the ileum, and proximal and distal colon compartments. The microbial function and composition were continuously monitored for 3 weeks (d35–d56) without altering the physiological conditions applied to the bioreactors, thereby maintaining eubiosis conditions.

Any changes observed in the microbiome between the baseline and eubiosis study periods were attributed to the introduction of the five fungal species. Additionally, the study assessed the ability of these fungal species to colonize and thrive in different intestinal regions under eubiosis conditions. To account for potential interindividual differences in microbial composition, the study involved three healthy adult participants. The experimental design is summarized in Fig. 1(B) and the experimental timeline in Fig. 1(C).

Throughout the experiments, samples for metabolic, microbial, and flow cytometry analysis were collected before the start of a new feeding cycle, hence during the stationary phase of microbial growth.

#### Dysbiosis study to elucidate the ability of five fungal species to colonize an impaired microbial community

The dysbiosis study aimed to assess the ability of five distinct fungal species to colonize the gut microbiome under dysbiosis conditions, which were induced by antibiotic treatment.

Briefly, following the initial period of eubiosis conditions (d35–d56), clindamycin was administered in the ileum compartments. A clindamycin hydrochloride stock solution was prepared twice a week at 3.15 g l^−1^ in water, filter-sterilized (0.2 µm) into an autoclaved Schott bottle, and sparged with N_2_ to ensure anaerobicity. The solution was pumped automatically into the ileum compartments every 8 h, for a duration of 14 days (d56–d70). Clindamycin reached a final concentration of 113.4 mg l^−1^ in the ileum compartments in *ca*. 3 days, and 85.0 mg l^−1^ in the proximal and distal colon compartments, in *ca*. 6 days. Recognizing that the five fungal species might face challenges in colonizing the microbiome under eubiosis conditions, they were reintroduced once daily for three consecutive days (d63–d65) after 1 week of antibiotic administration.

The first week of antibiotic treatment, before the reinoculation of the fungal species, aimed to confirm fungal engraftment that may have occurred during eubiosis conditions. Opportunistic fungi that managed to thrive under eubiosis conditions might exploit the reduced bacterial function and diversity induced by antibiotics, resulting in higher cell concentrations. In contrast, the second week of the antibiotic treatment, following the reinoculation of the five fungal species, was used to determine whether any of these species, which might not have thrived under eubiosis conditions, could now colonize in the dysbiosed microbiome. Following the antibiotic cessation, the recovery of the microbial ecosystem was observed for one and a half weeks (d70–d79). This phase aimed to assess the competitive ability of opportunistic fungi against a recovering colonic microbiome. The experimental timeline is described in Fig. 1(C).

Throughout the experiments, samples for metabolic, microbial, and flow cytometry analysis were collected before the start of a new feeding cycle, hence during the stationary phase of microbial growth.

### Fungal inoculum preparation and inoculation

*Candida albicans* strain SC5314 (patient with disseminated candidiasis) (Odds et al. 2004) was provided by the research group of Dr Salomé LeibundGut-Landmann (University of Zürich, Zürich, Switzerland). *Candida tropicalis* strain IHEM6204 (human sputum from a patient with disseminated aspergillosis and lung cancer), *N. glabratus* strain IHEM19218 (human blood from a patient with candidemia and prostate cancer), *C. parapsilosis* strain IHEM17737 (human faeces), and *S. cerevisiae* strain IHEM17798 (human faeces from a patient with fungemia and stubborn anemia) were purchased from the BCCM/IHEM fungi collection (Sciensano, Brussels, Belgium). Upon receival, each fungal species identity was confirmed by Sanger sequencing.

Each fungal species was initially cultured separately on Sabouraud dextrose agar (Avantor™) for 48 h under anaerobic conditions, using an AnaeroGen™ bag, at room temperature. Subsequently, a single colony was selected to inoculate 20 ml Sabouraud dextrose broth (2% w/v), and this culture was further incubated under anaerobic conditions within an anaerobic chamber at 37°C, for 48 h to prepare the first inoculum (to be used on day 35 or 63). However, after 24 h of incubation, 400 µl of each overnight culture was used to inoculate distinct 20 ml fresh Sabouraud dextrose broth (2% w/v), and this was also incubated anaerobically for 48 h (to be used on day 36 or 64). This process was repeated once more to prepare the third inoculum (to be used on day 37 or 65). Therefore, only fresh cultures were used to prepare the different inocula.

To prepare those inocula, equal volume of the cultures from each species were pooled in a 100 ml-clear glass vial (reference A05521, Novolab, Geraardsbergen, Belgium) closed with a butyl stopper (reference 7395, Rubber B.V., Loosdrecht, The Netherlands) and a crimp cap (reference 548–3317, Avantor™) that had been preflushed with N_2_ to ensure the preservation of an anaerobic environment. After homogenizing the mixture, 5 ml was withdrawn using a syringe mounted with a needle, and this inoculum was used to inoculate each intestine compartment once a day (on days 35–37 and days 63–65) after the feeding cycle was completed. The headspace of the ileum, proximal colonic, and distal colon compartments was flushed with N_2_ immediately after fungal inoculation.

### Faecal sample collection and donor description

The three healthy adults had no antibiotic nor pre- or probiotic dietary supplements intake in the previous 3 months, and no constipation. All faecal samples were immediately transferred to a recipient containing an AnaeroGen™ bag to limit the samples’ exposure to oxygen, and transferred to the lab for further use. The proximal and distal colon compartments were inoculated within maximum 13 h postcollection of the faecal samples by the donors.

### Metabolic analysis

To assess the impact of the fungal inoculation under eubiosis and dysbiosis conditions on the metabolic activity of the gut microbiome, lactate, ethanol, SCFA (acetate, propionate, and butyrate), and branched short-chain fatty acid (BSCFA, isobutyrate, isovalerate, and isocaproate) were quantified at all sampling time points.

Lactate was quantified on cell-free fermentation samples (centrifuged for 5 min at 7690 × *g*) using a commercially available enzymatic assay kit (R-Biopharm, Darmstadt, Germany) according to the manufacturer’s instructions.

Ethanol, SCFA, and BSCFA were quantified using a GC-2030 gas chromatograph (Shimadzu, Hertogenbosch, Netherlands). It was equipped with a GC SH-polarD capillary column (30 mm × 0.32 mm ID-BP 21 × 0.25 µm, Shimadzu), a flame-ionization detector, and a split injector. Nitrogen (90.3 ml min^−1^) was used as a carrier gas; nitrogen (24 ml min^−1^), hydrogen (32 ml min^−1^), and air (200 ml min^−1^) were used as make-up gas. The injector and detector temperatures were set at 200°C and 240°C, respectively. The column temperature profile was set from 40°C to 220°C with a constant temperature increase of 10°C min^−1^ after which temperature was held for 5 min at 220°C. Fermentation samples collected during the Mucosal Ileum-SHIME® experiment were diluted 1:1 with a mixture composed of acetonitrile, 0.5% (v/v) formic acid, 0.008% (v/v) 2-methyl hexanoic acid (internal standard), and a saturating amount of sodium chloride; mixed for 15 min using a HulaMixer™ Sample Mixer (ThermoFisher Scientific, MA, USA); centrifuged (15 080 × *g*, 15 min); and run with the appropriate external standards. Injection was performed in split mode with a split ratio of 40; the injected volume was 1 µl.

All analysis were performed in simplicate. Due to limited concentration of BSCFA (Supplementary Raw data), only lactate, ethanol and SCFA data are reported in the manuscript.

### Live bacterial cell count quantification by flow cytometry

Live bacterial cells were quantified *via* flow cytometry to evaluate the impact of the five fungal species inoculation on bacterial survival in eubiosis and dysbiosis. Samples were 1:1-diluted in cryoprotectant and stored at –80°C until analysis as previously described (Hoefman et al. 2012). Upon staining with 0.01 mM SYTO24 (ThermoFisher Scientific) and 0.0 mM propidium iodide (ThermoFisher Scientific) at room temperature for 15 min in the dark, samples were analysed on a BD Facsverse (BD, Franklin Lakes, NJ, USA) using the high flowrate setting. Flow cytometry data were analysed using FlowJo version 10.5.0. (BD).

### Microbial community analysis by qPCR

Total bacterial and species-specific concentrations were determined by quantitative polymerase chain reaction (qPCR) on all time points of the experiment, on the three faecal inocula, and the different fungal overnight cultures. Briefly, DNA was isolated as previously described (Boon et al. 2003) with minor modifications (Duysburgh et al. 2019) from 1 ml luminal samples or 0.25 g of faecal matter. Subsequently, qPCR was performed using a QuantStudio 5 Real-Time PCR system (Applied Biosystems, Foster City, CA, USA). Each sample was run in technical triplicate.

Total bacterial concentration determination was performed as previously described (Marsaux et al. 2023) with the primers UNI-F (5′-GTGSTGCAYGGYYGTCGTCA-3′) and UNI-R (5′-ACGTCRTCCMCNCCTTCCTC-3′) (Maeda et al. 2003), which target the 16S rRNA gene. Results are reported as log_10_(16S rRNA gene copies ml^−1^).

*Candida albicans* quantification was performed using the primers (0.3 µM) Calb-F (5′-GGGTTTGCTTGAAAGACGGTA-3′) and Calb-R (5′-TTGAAGATATACGTGGTGGACGTTA-3′) (Guiver et al. 2001), the probe (0.2 µM) Calb-P (5′-FAM-ACCTAAGCCATTGTCAAAGCGATCCCG-3′) (Guiver et al. 2001), and the SensiFast™ Probe Low-ROX kit (Bioline GBmH, Luckenwalde, Germany). After an initialization step at 95°C for 5 min, 40 cycles of a denaturation step at 95°C for 10 s followed by an annealing step at 60°C for 30 s were performed at a ramp rate of 1.6°C s^−1^.

*Candida tropicalis, N. glabratus, C. parapsilosis*, and *S. cerevisiae* quantifications were all performed similarly. Briefly, 0.3 µM of both species-specific forward and reverse primers were used with the SensiMix™ SYBR® Low-ROX kit (Bioline GBmH). After an initialization step at 95°C for 10 min, 40 cycles of a denaturation step at 95°C for 15 s followed by an annealing step at 60°C for 30 s and an elongation step at 72°C for 30 s were performed at a ramp rate of 1.6°C s^−1^. After the cycles ended, a melting curve analysis was performed with a dissociation step from 60°C to 95°C, and a ramp rate of 0.075°C s^−1^. The primers used are as follows: *C. tropicalis* Ctrop-F (5′-AATCCGAAGGCTTGATGG-3′) and Ctrop-R (5′-AATGCCAGCAGCAAAAGTAG-3′) (Rajasekharan et al. 2018), *C. parapsilosis* Cpara-F (5′-ATTTTGTATGTTACTCTCTCG-3′), and Cpara-R (5′-TGCCAACATCCTAGGCCGAAGC-3′) (Ogata et al. 2015), *N. glabratus* Cgla-F (5′-GTGAAAGTTTCGTTGCTG-3′) and Cgla-R (5′-TGGGCTGAAATGTTGGAC-3′) (Kumar and Ramesh 2014), and *S. cerevisiae* Scere-F (5′-AGGAGTGCGGTTCTTTG-3′) and Scere-R (5′-TACTTACCGAGGCAAGCTACA-3′) (Chang et al. 2007).

The standards used were synthesized gBlock Gene Fragments (Integrated DNA Technologies, Inc., Coralville, IA, USA) of 1000 bp designed by aligning the primer pairs on the reference genome of the species of interest, which was obtained from NCBI (Bethesda, MD, USA). For the total bacteria standard, *Escherichia coli* strain 97–3250 was used. The complete sequence of each standard can be found in the Supplementary Raw data.

To better appreciate the differences in concentration between each fungal species, qPCR values were corrected using flow cytometry data. To do so, four to six pure overnight cultures of each fungal species were analysed both by qPCR (triplicate of analysis) and by flow cytometry (simplicate of analysis). The average quotient of the gene copy number and the active fluorescent unit (afu) count was used as a correction factor of the qPCR data obtained for each fungal species present in a mixed community (faecal or luminal samples). Therefore, the qPCR data for each fungal species are expressed in log_10_ (afu ml^−1^).

### Bacterial community analysis by 16S rRNA gene-targeted sequencing

Bacterial community composition was assessed on a selection of time points. Samples were sent out to LGC Genomics GmbH (Berlin, Germany) for Illumina sequencing of the 16S rRNA gene amplicons of the V3–V4 region. The 341F (5′-CCTACGGGNGGCWGCAG-3′) and 785R (5′-GACTACHVGGGTATCTAAKCC-3′) primers were used (Herlemann et al. 2011). The amplification by PCR, sequencing of the amplicons, and preparation of the Illumina libraries were performed as previously described (Marsaux et al. 2023).

### Data analysis

In the case of total bacterial, species-specific and cell count concentrations, data were log-transformed because of their log-normal distribution.

The impact of the fungal inoculation on the change in total bacterial, species-specific, cell count, and metabolic concentrations was assessed using a Kruskal–Wallis test followed by Dunn’s multiple comparison test. During the eubiosis study, the outcomes were compared for change between the baseline period (average of d19–d35), the week 1 of the eubiosis period (d36–d42), and the weeks 2 and 3 of the eubiosis period (d44–d56). During the dysbiosis study, the outcomes were compared for change between the week 3 of the eubiosis period (d51–d56), the first week of the dysbiosis period (d58–d63), the second week of the dysbiosis period (d64–d70), and the recovery period (d71–d79). Statistical analysis was performed using GraphPad Prism version 9.5.1 (733) for Windows (GraphPad Software, San Diego, CA, USA).

Read assembly and cleanup was largely derived from the MiSeq standard operating procedure described by the Schloss lab (Schloss and Westcott 2011, Kozich et al. 2013). In brief, the Mothur software package (v.1.44.3) was used to assemble reads into contigs, perform alignment-based quality filtering (alignment to the Mothur-reconstructed SILVA SEED alignment, v.138), remove chimeras (vsearch v2.13.3), assign taxonomy using a naïve Bayesian classifier (Wang et al. 2007) and SILVA NR v138, and cluster contigs into OTUs at 97% sequence similarity. Only sequences having at least 5 counts in one sample and assigned to the bacteria kingdom were retained. For each OTU, representative sequences were picked as the most abundant sequence within that OTU.

Data at genus level was processed after applying a total sum normalization. To account for variations in total bacterial cell counts among samples, the relative abundances of bacterial genera was corrected with 16S rRNA qPCR data, enabling the generation of quantitative microbiome profiles. Additionally, the gut bacteriome α-diversity was assessed by using Shannon’s and Simpson’s indexes (calculated using phyloseq, v1.44.0; McMurdie and Holmes 2013), while the β-diversity was visualized through discriminant analysis of principal components (DAPC), principal component analysis (PCA), and redundancy analysis (RDA) based on the relative abundances of bacterial OTU. Specifically, the DAPC was conducted by hierarchically clustering Euclidean distances between samples using Ward’s minimum variance method. The DAPC plots were constructed with two discriminants and 80% of retained variance in the principal components using adegenet (v2.1.10) (Jombart 2008, Jombart et al. 2010). The RDA was performed with the taxonomic relative abundances of bacterial OTU as a response variable and the treatment as explanatory variable using type 2 scaling. The RDA model and its statistical significance were calculated in vegan (v2.6–4). DAPC and RDA plots were visualized using ggplot2 (v3.4.3). PCA were performed on normalized data of the 16S rRNA gene-targeted sequencing using ClustVis (https://biit.cs.ut.ee/clustvis/, 19 July 2023, date last accessed) with parameters as previously described (Van den Abbeele et al. 2021).

## Results

### Quantification of the five fungal species in the donor faecal samples and the fungal inocula used in the eubiosis and dysbiosis studies

The concentrations of *C. albicans, C. parapsilosis, C. tropicalis, N. glabratus*, and *S. cerevisiae* were determined by qPCR in the three faecal samples and the fungal inocula used in the eubiosis and dysbiosis studies.

**Figure 2.**
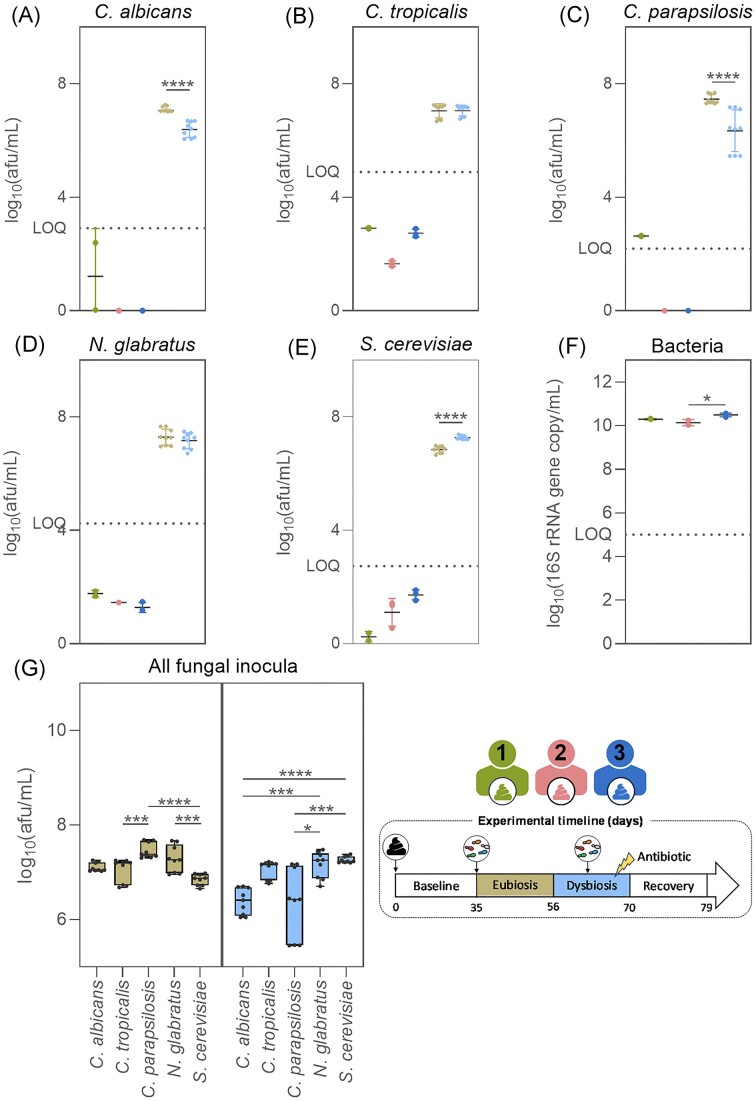
*Candida albicans* (A), *C. tropicalis* (B), *C. parapsilosis* (C), *N. glabratus* (D), *S. cerevisiae* (E), and total bacteria concentrations (F) in the faecal samples from donor 1, 2, or 3, and in the fungal inocula used during the eubiosis study at days 35–37 and the dysbiosis study at days 63–65 (G). Box plots are box and whiskers showing minimum and maximum values. Significant differences were assessed using Mann–Whitney tests for comparisons between the fungal inocula used in the two study periods, Kruskal–Wallis tests with Dunn’s multiple comparisons for differences between fungal species within inocula, and Kruskal–Wallis tests with Dunn’s multiple comparisons for total bacteria concentrations between the three faecal samples. Significant differences are marked with asterisks (*P < .05, **P < .01, ***P < .001, and ^****^P < .0001).

For the three faecal samples, all five fungal species displayed levels below the limit of quantification, as indicated by all qPCR analyses (Fig. 2A–E).

The three fungal inocula used in the eubiosis study (d35–d37) contained a significantly higher quantity of *C. parapsilosis* cells compared to *C. tropicalis* and *S. cerevisiae* (Fig. 2G). Conversely, during the dysbiosis study (d63–d65) the inocula contained a significantly higher concentration of *S. cerevisiae* and *N. glabratus* cells than *C. albicans* and *C. parapsilosis* (Fig. 2G). In addition, the inocula used in the eubiosis study contained significantly more *C. albicans, C. parapsilosis*, and *S. cerevisiae* cells compared to the inocula used in the dysbiosis study (Fig. 2A–E). This discrepancy may lead to species-specific differences within the SHIME® reactors between the different study periods. Regardless, all fungal species were present in high enough concentrations in the inocula to obtain values above the limit of quantification in the respective SHIME® reactors upon inoculation.

Candida species-specific colonization results in reduced SCFA productions by the colonic microbiota without altering the bacterial composition and diversity in eubiosis conditions

#### Cell concentrations of the five fungal species and total bacteria in eubiosis conditions

**Figure 3.**
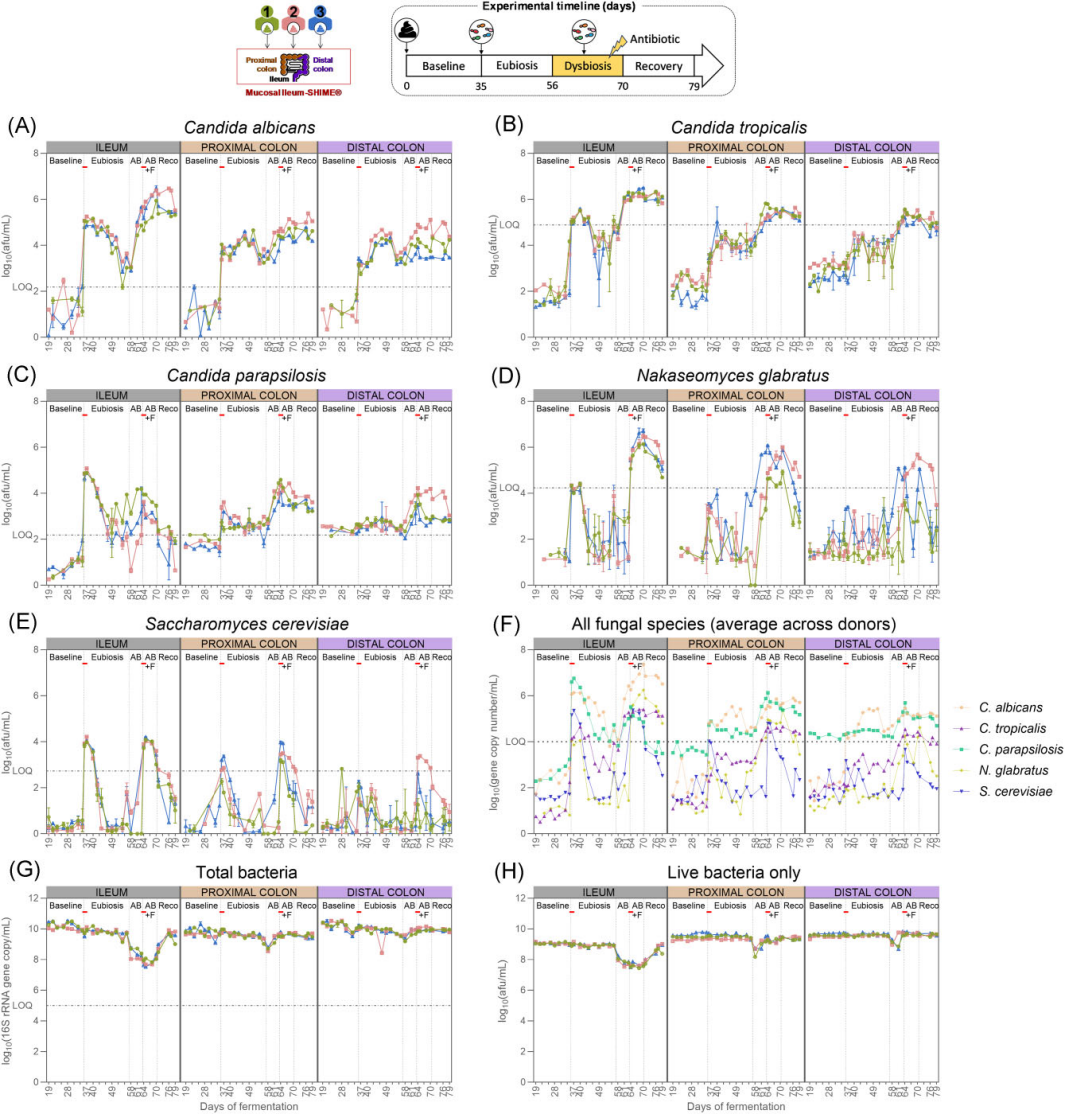
*Candida albicans* (A), *C. tropicalis* (B), *C. parapsilosis* (C), *N. glabratus* (D), *S. cerevisiae* (E), and total bacteria concentrations (G) measured by qPCR (*n* = 3) (A) and total live bacteria concentrations measured by flow cytometry (*n* = 1) (H) in the ileum, and the proximal and distal colon compartments inoculated at day 0 with the faecal samples from donor 1, 2, or 3. The fungal species values were corrected with flow cytometry data obtained on their respective pure overnight culture, hence resulting in cell counts instead of gene copy counts. The uncorrected average values across the three donors for each intestinal region and each species is also provided (F). Samples were collected during the baseline period (d19–d35), during the eubiosis study (d36–d56) following the fungal inoculations for three consecutive days (d35–d37), during the first week of clindamycin treatment (d56–d63; AB = antibiotic), during the second week of clindamycin treatment (d63–d70; AB+F = antibiotic with fungal reinoculation), which started with the reinoculation of the fungal species for three consecutive days (d63–d65), and during the recovery period (d70–d79; Reco = recovery). The fungal (re-) inoculations are indicated by an horizontal line. The limit of quantification (= LOQ) is indicated by an horizontal dotted line.

The concentrations of *C. albicans, C. tropicalis, N. glabratus, C. parapsilosis, S. cerevisiae*, and total bacteria were determined in the SHIME® using qPCR (Fig. 3A–G), and the live bacteria concentrations were quantified by flow cytometry (Fig. 3H) at all sampling time points during the eubiosis study. Detailed statistical analysis for each fungal species is provided in Fig. S1(A–E). Similar results were observed during the three experiments despite the fact that different donors were used to inoculate the colon compartments.

As expected from the low initial levels of the five fungi present in the respective faecal inocula, none of the intestinal regions in the SHIME® contained levels of the fungal species above the limit of quantification during the baseline period (Fig. 3A–E). Subsequently, upon inoculation of the fungal strains, all fungal species concentrations significantly increased, particularly in the ileum compartments. In the second- and third-week postinoculation, concentrations of *C. tropicalis, N. glabratus*, and *S. cerevisiae* significantly decreased, reaching levels below the limit of quantification. Conversely, *C. albicans* and *C. parapsilosis* maintained relatively high cell counts, eventually reaching similar concentrations in all intestinal compartments.

Importantly, the engraftment success is independent of inoculation density: *N. glabratus* did not persist in the bioreactors despite the fact its inoculum had, on average, one of the largest concentration of cells (Fig. 2G).

Lastly, the total bacterial concentration gradually and significantly decreased in all intestinal regions between the baseline period and the second- and third-weeks postinoculation (Fig. 3G, Fig. S1F). For live bacteria specifically, it was only in the ileum compartment that there was a significant decrease, as quantified by flow cytometry (Fig. 3H, Fig. S1G).

#### Impact of the fungal inoculation on the metabolic activity of the gut microbiome in eubiosis conditions

**Figure 4.**
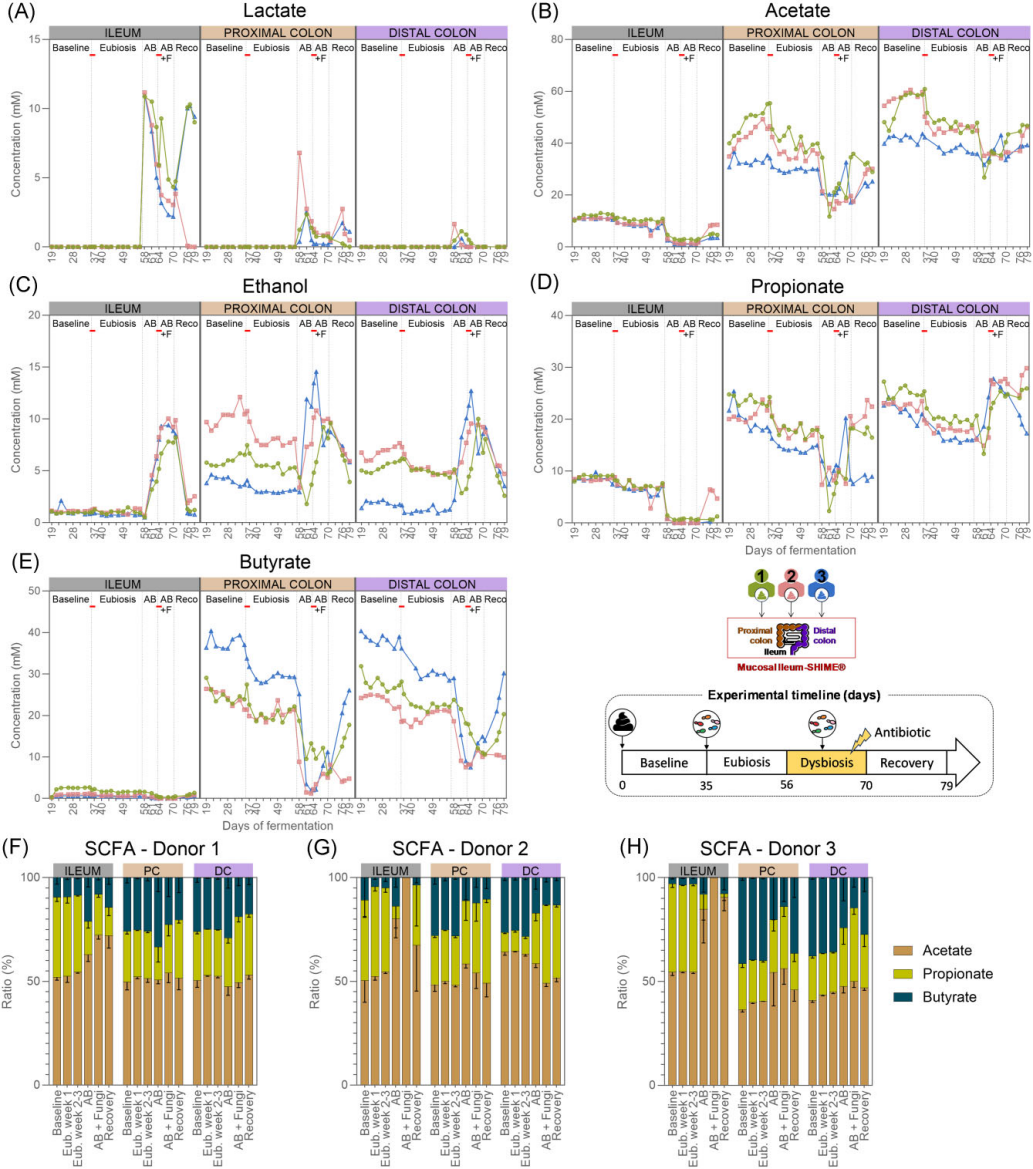
Lactate (A), acetate (B), ethanol (C), propionate (D), butyrate concentrations (E) (*n* = 1), and SCFAs ratios (mean ± standard deviation) (F–H) in the ileum, and the proximal and distal colon compartments inoculated at day 0 with the faecal samples from donor 1, 2, or 3. Samples were collected during the baseline period (d19–d35), during the eubiosis study (d36–d56, Eub = eubiosis) following the fungal inoculations for three consecutive days (d35–d37), during the first week of clindamycin treatment (d56–d63; AB = antibiotic), during the second week of clindamycin treatment (d63–d70; AB+F = antibiotic with fungal reinoculation), which started with the reinoculation of the fungal species for three consecutive days (d63–d65), and during the recovery period (d70–d79; Reco = recovery). The fungal (re-) inoculations are indicated by an horizontal line. PC = proximal colon; DC = distal colon.

The metabolic activity of the ileal microbiome remained largely stable between the baseline period and the eubiosis period following fungal inoculations, with the exception of lactate and acetate (Fig. 4A–E, Fig. S2A–E). Lactate values strongly increased in the ileum compartment 1 but remained exceptionally low (<0.06 mM). Acetate and propionate concentrations gradually declined in all ileum compartments, and values were significantly lower during the second- and third-weeks postinoculation compared to the baseline period. Despite these changes, the acetate:propionate:butyrate ratio remained stable throughout the experiment (Fig. 4F–H).

In contrast, in each colon region, all metabolic markers significantly decreased between the baseline period and the period following the fungal inoculations, except for lactate, which remained unchanged (Fig. 4A–E, Fig. S2A–E). Despite a significant reduction in acetate, propionate, and butyrate, the acetate:propionate:butyrate ratio remained stable throughout the experiments, regardless the donor that was used (Fig. 4F–H). Furthermore, ethanol concentrations also significantly decreased. Detailed statistics for each metabolite are provided in Fig. S2(A–E).

#### Impact of the fungal inoculation on the gut bacteriome composition and diversity in eubiosis conditions

**Figure 5.**
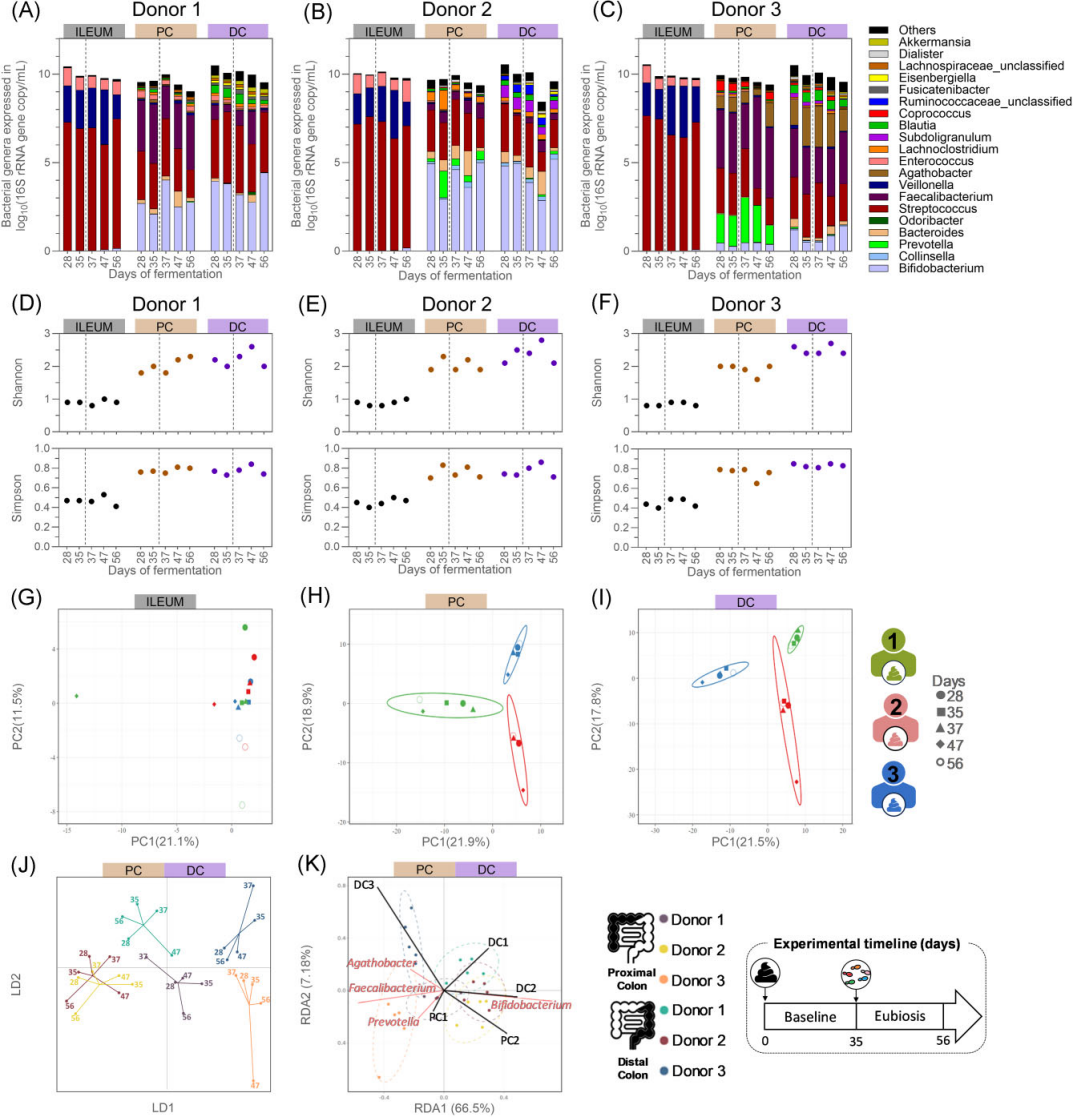
Quantitative microbiome profiling from the ileum inoculated with a bacterial consortium, from the proximal colonic, and distal colonic compartments inoculated with faecal inoculum of donor 1 (A), 2 (B), or 3 (C), and their resulting bacterial α-diversity indexes (D–F) and β-diversity (G–K), from samples collected during the baseline and eubiosis periods. (A–F) The dotted lines separate the baseline period from the eubiosis one, which starts with the inoculation of the fungal species for three consecutive days (d35–d37). (A–C) Each bar represents the total bacteria concentration in the sample, whereas the colours represent the proportion of each bacterial genera. Gut bacteriome β-diversity based on operational taxonomic unit (OTU) relative abundances and analysed through separated PCA for all ileum (G), proximal (H), and distal (I) colon compartments, and through DAPC (J) and RDA (K) for all colon compartments only. PC = proximal colon; DC = distal colon; DAPC = discriminant analysis of principal component; LD = linear discriminant; RDA = redundancy analysis; PCA = principal component analysis; and PC (*x*- and *y*-axis of PCA plot) = principal component.

The composition of the gut bacteriome remained stable in all intestinal regions between the baseline period and the period following fungal inoculation in eubiosis conditions (Fig. 5A–K, Table S1). In the ileum compartments, *Streptococcus, Veillonella*, and *Enterococcus* were dominant throughout the eubiosis experiment (Fig. 5A–C). Similarly, the ratios between the dominant phyla remained stable in the proximal, and, albeit to a lesser extent, in the distal colon compartments (Table S1). At genus level, temporal changes in relative abundance were limited. Supporting these observations, the α-diversity indexes of the gut bacteriome remained stable throughout all time points in the ileum compartments, while limited temporal variations were observed in the colonic regions (Fig. 5D–F). Both the Shannon’s and the Simpson’s indexes were similar on days 28 and 56 in most colon compartments. Furthermore, the analysis of the gut bacteriome β-diversity showed that samples tended to cluster by donor and intestinal regions, maintaining donor-specific features (Fig. 5G–K). Notably, samples collected on days 28 and 56 showed close clustering in all colon compartments.

Overall, these data demonstrated that distinct microbial communities were established in each intestinal compartment, and that the interindividual differences were maintained between donors. Hence, our observations suggest that the inoculation of the five different fungal species into a wide range of donor- and region-specific microbiota had no impact on the overall bacterial composition.

### Altering the gut bacteriome composition and diversity with antibiotics enables higher fungal engraftment, with donor-, species-, and intestinal region-dependent variations

#### Cell concentrations of the five fungal species and total bacteria in impaired conditions

The antibiotic treatment significantly impacted total bacterial concentrations in the ileum compartments but had a minor effect on the colon compartments, regardless of the donor used during the experiment (Fig. 3G–H, Fig. S1F–G). Upon cessation of antibiotic administration, bacterial concentrations significantly decreased in the proximal colon compartments, remained stable in the distal colon compartments, but significantly increased in the ileum compartments. Similar patterns were observed by flow cytometry, with the antibiotic treatment mostly impacting the ileum compartments (Fig. 3H).

With respect to the fungal species, only *C. tropicalis* concentrations showed significant increases during the first week of antibiotic treatment in the ileum compartments, while *N. glabratus* and *S. cerevisiae* remained below the limit of quantification (Fig. 3A–F, Fig. S1A–E). Conversely, *C. albicans* levels increased, albeit not significantly, while C*. parapsilosis* levels remained stable, displaying variability among ileum compartments.

In the colon compartments, the fungal concentrations were donor specific. *Candida parapsilosis, C. tropicalis*, and *N. glabratus* concentrations significantly increased across all three proximal colon compartments, although *N. glabratus* remained below the limit of quantification in donors 1 and 2, and *C. tropicalis* in donor 3. *Candida albicans* concentrations also increased in all three proximal colon compartments, albeit not significantly. *Saccharomyces cerevisiae* levels remained stable and below the limit of quantification. In contrast, no changes were observed in the distal colon compartment from donor 1. In the one from donor 2, concentrations of *C. albicans, C. parapsilosis, C. tropicalis*, and *N. glabratus* increased, while only *C. tropicalis* and *N. glabratus* increased in the compartment from donor 3. Despite these increases, values remained below the limit of quantification for *C. tropicalis* in both donors 2 and 3, and for *N. glabratus* in donor 2.

During the second week of antibiotic treatment, the reinoculation of the five fungal species led to a significant increase in *C. albicans, C. tropicalis*, and *N. glabratus*, while *C. parapsilosis* decreased. Every fungal species significantly increased in the proximal colon compartments, albeit not significantly for *C. parapsilosis*. On the other hand, *C. parapsilosis, C. albicans*, and *C. tropicalis* concentrations significantly increased in the distal colon compartments, while *S. cerevisiae* and *N. glabratus* concentrations remained below the limit of quantification, except for donor 2. *Candida albicans* levels especially increased in the distal colon compartment of donor 2.

During the recovery period, *C. albicans, N. glabratus*, and *C. tropicalis* concentrations remained stable across all intestinal regions. *Candida parapsilosis* significantly decreased in the ileum compartments, decreased in the proximal compartments, and remained stable in the distal colon compartments. *Saccharomyces cerevisiae* concentrations decreased significantly in all intestine compartments, reaching values below the limit of quantification. Importantly, *C. parapsilosis* was the only fungal species that reached higher levels in the proximal and distal colon compartments compared to the ileum compartments by the end of the experiment for all three donors.

Finally, the engraftment success is independent of inoculation density: *C. albicans* persisted in the bioreactors despite the fact its inocula had, on average, the lowest amount of cells. In contrast, *N. glabratus* and *S. cerevisiae* inocula contained significantly more cells and did not persist (Fig. 2G). Detailed growth statistics for each fungal species and total bacteria are provided in Fig. S1(A–F).

#### Metabolic activity of the gut microbiome in impaired conditions

The metabolic activity of the ileal microbiome was profoundly affected by the antibiotic treatment (Fig. 4A–E, Fig. S2A–E). Acetate and propionate, but not butyrate, concentrations significantly decreased in the ileum compartments after 1 week of antibiotic treatment. These concentrations remained stable for the remainder of the experiment, except in the ileum compartment 2 in which the concentrations returned to levels comparable to the ones before treatment. These results suggest that the fungal reinoculation had limited impact on the metabolic activity of the ileum microbiome, compared to the antibiotic treatment. This initial reduction influenced the acetate:propionate:butyrate ratio, shifting it by the end of the experiment towards increased acetate and butyrate in the ileum compartments 1 and 3, and acetate only in the ileum compartment 2 (Fig. 4F–H). The reduced production of SCFAs resulted in an accumulation of lactate due to reduced cross-feeding activity. Indeed, lactate concentrations significantly increased at the start of the antibiotic treatment, and although they decreased by the end of the experiment, they remained elevated. Ethanol concentrations only significantly increased once the fungal species were reintroduced and returned to the original level upon discontinuation of the antibiotic administration.

The metabolic activity of the colon microbiome was significantly impacted by the antibiotic treatment (Fig. 4A–E, Fig. S2A–E). In the proximal colon compartments, butyrate and acetate significantly decreased after 1 week of antibiotic treatment. Although propionate concentrations decreased, this reduction was not statistically significant. Similar, though nonsignificant, decreases in acetate and butyrate, but not propionate, were observed in the distal colon compartments. The SCFAs level remained largely similar in all colon compartments during the second week of antibiotic treatment, except propionate, which significantly increased in the distal colon compartments. After ceasing antibiotic administration, levels in acetate, propionate, and butyrate changes were donor dependent. Acetate, propionate, and butyrate concentrations increased in both colon compartments from donor 1. For donor 2, acetate reincreased in both colon compartments, while propionate only reincreased in the proximal colon compartment. Finally, only butyrate reincreased in both colon compartments from donor 3. Consequently, the acetate:propionate:butyrate ratio strongly shifted in the colon compartments from donors 2 and 3 but not donor 1 (Fig. 4F–H). These changes did not result in lactate accumulation; instead, lactate only significantly increased during the first week of antibiotic administration but returned to comparable levels by the end of the experiment. Additionally, while ethanol concentration significantly increased upon the reintroduction of the fungal species, it returned to comparable levels after antibiotic cessation.

#### Impact of the antibiotic treatment on the gut bacteriome composition and diversity

**Figure 6.**
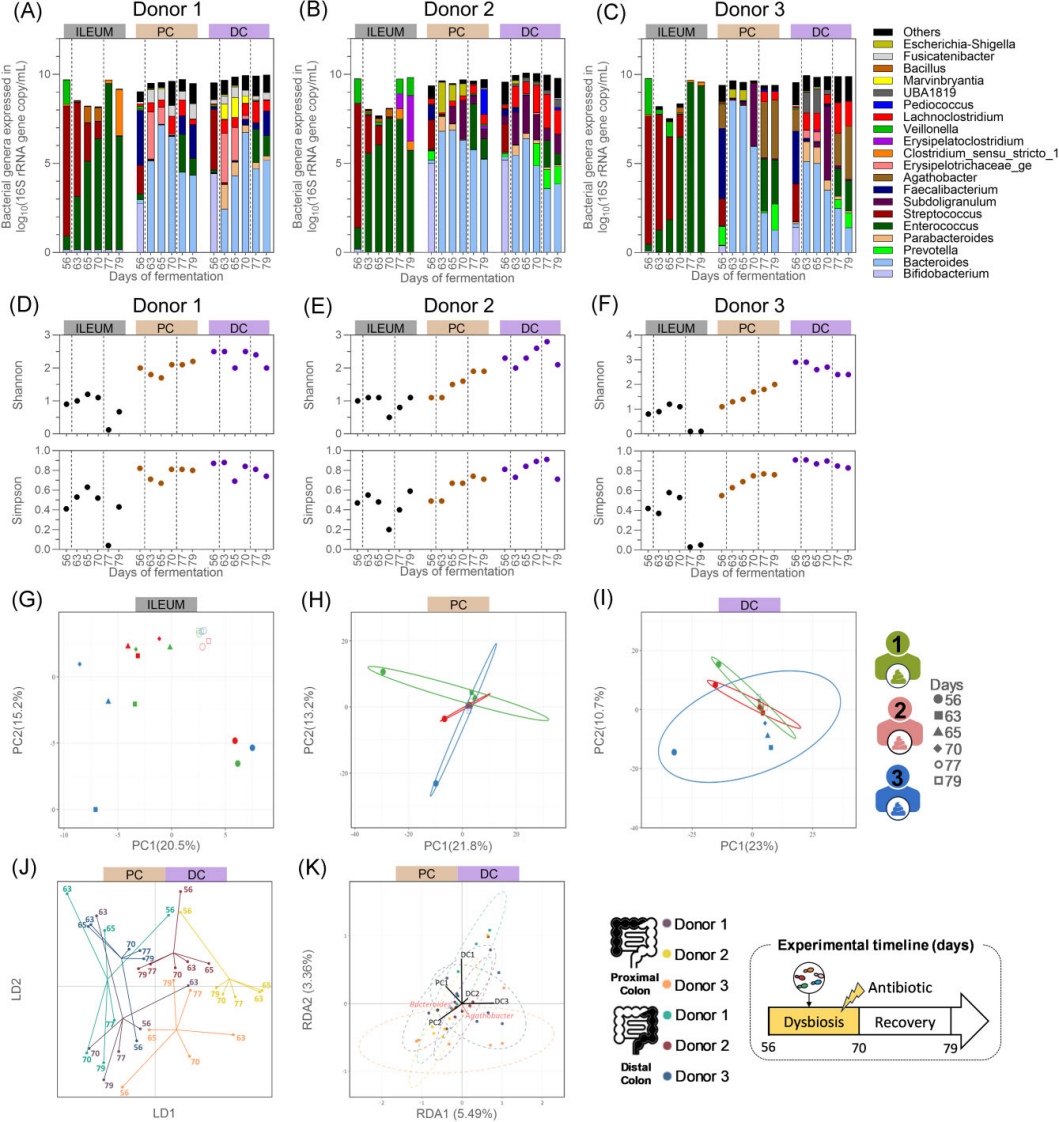
Quantitative microbiome profiling from the ileum inoculated with a bacterial consortium, from the proximal colonic, and distal colonic compartments inoculated with faecal inoculum of donor 1 (A), 2 (B), or 3 (C), and their resulting bacterial α-diversity indexes (D–F) and β-diversity (G–K), from samples collected during the antibiotic treatment and the recovery periods. (A–F) The left dotted lines separate the end of the eubiosis period from the antibiotic treatment during which the five fungal species were reintroduced in the bioreactors (d63–d65), and the right dotted lines the end of the antibiotic treatment and the start of the recovery period. (A–C) Each bar represents the total bacteria concentration in the sample, whereas the colours represent the proportion of each bacterial genera. Gut bacteriome β-diversity based on OTU relative abundances and analysed through separated PCA for all ileum (G), proximal (H), and distal (I) colon compartments, and through DAPC (J) and RDA (K) for all colon compartments only. PC = proximal colon; DC = distal colon; DAPC = discriminant analysis of principal component; LD = linear discriminant; RDA = redundancy analysis; PCA = principal component analysis; and PC (*x*- and *y*-axis of PCA plot) = principal component.

The gut bacteriome was substantially impacted by the antibiotic treatment in all intestinal regions (Fig. 6A–K). In the ileum compartments, the microbiome shifted from a predominance of *Streptococcus* to a dominance of *Enterococcus* by the end of the antibiotic treatment (Fig. 6A–C). As a result, the Shannon’s and Simpson’s indexes decreased either by the end of the antibiotic period (ileum compartment 2) or at the beginning of the recovery period (ileum compartments 1 and 3) (Fig. 6D–F). By the end of the experiment, comparable index values were observed compared to the preantibiotic treatment levels in the ileum compartments 1 and 2, while the indexes remained low in the ileum compartment 3. Despite these variations, two distinct clusters of samples were observed, with samples collected on days 77 and 79, after cessation of the antibiotic treatment, on one hand, and those from day 56, before initiation of the antibiotic treatment, on the other (Fig. 6G), demonstrating the consistent and long-lasting effect of clindamycin on the ileal microbiome.

The ratios between the dominant phyla also shifted in the colon compartments, resulting in the dominance of *Bacteroides*, at genus level, regardless of the donor used (Fig. 6A–C). The colon bacteriome α-diversity was more impacted in the proximal colon compartments compared to the distal ones (Fig. 6D–F). While both the Shannon’s and Simpson’s indexes decreased in the colon compartments from donor 1, to then return to their initial levels by the end of the experiment, they steadily increased in the proximal colon compartments from donors 2 and 3. Despite these variations, all the samples collected during the antibiotic treatment and recovery clustered closely together, distinct from the samples collected before the initiation of antibiotic treatment (Fig. 6H–K). This was associated with a loss of donor-specific features, as observed on the RDA plot which only account for 9% of explanatory variations on the first two dimensions (RDA1 and RDA2) of the ordination space (Fig. 6K).

## Discussion

In the eubiosis study, we investigated the colonization potential of *C. albicans, C. tropicalis, N. glabratus*, and *C. parapsilosis* within the Mucosal Ileum-SHIME® model, alongside *S. cerevisiae*, a transient microbe of the human gastrointestinal tract (Auchtung et al. 2018). Despite their global prevalence (d’Enfert et al. 2021, Delavy et al. 2022), none of those fungal species were detected in the faecal samples used for inoculation. Even if they would be present (at level below the limit of detection), their niche occupancy will have a negligible impact compared to that of the highly inoculated fungi.

Upon inoculation, the five fungi exhibited a remarkably similar behaviour across the different donors’ SHIME®, although colonized by distinct colonic microbiomes. Notably, *S. cerevisiae* did not persist in the model, serving as a control to confirm that artificial inoculation with highly concentrated species does not enable transient microbes to engraft the microbiome. In addition, *C. albicans* and *C. parapsilosis* colonized all intestine compartments whereas *C. tropicalis* and *N. glabratus* concentrations remained below the limit of quantification. This indicated that *Candida* could successfully colonize the gut microbiome under the applied eubiosis physiological conditions, yet in a species- (or strain-) dependent manner.

The absence of growth of *N. glabratus* and *C. tropicalis* may be attributed to intrinsic colonization capabilities or potential interspecies competition in the compartments. Notably, *C. albicans* (23.7%) and *C. parapsilosis* (10.7%) were the most common *Candida* species found in the faeces of healthy volunteers in the Human Microbiome Project, compared to *C. tropicalis* (6.5%) and *N. glabratus* (0.5%) (Nash et al. 2017). This suggests that some species may be better adapted to thrive in the human gastrointestinal tract of healthy individuals. Additionally, *C. albicans* exhibited the highest concentration among all *Candida* species across all intestinal regions, indicative of a competitive advantage. Previous studies have shown that *C. albicans* demonstrates considerable flexibility in carbon utilization, a crucial requirement for surviving in the gastrointestinal tract where nutrient availability constantly fluctuates based on diet (Sandai et al. 2012). The adequate simulation of a diverse range of sugars in the ileal nutritional medium and fibers in the fiber solution used in our Mucosal Ileum-SHIME^®^ model may therefore specifically stimulate microbial species having a particular carbohydrate consumption flexibility, *in casu C. albicans*.

It is, however, noteworthy that the cell concentrations used for inoculating each fungal species were not uniform. These five fungal species were introduced into an established microbial community, which naturally offers colonization resistance (Lawley and Walker 2013). As a result, we reason that the ability of a fungal species to persist in any intestinal compartment does not depend on the inocula concentration but on inherent characteristics like adaptation to physiological, ecological, and environmental stressors within the existing microbial network and the SHIME^®^ model itself. Yet, the inoculum may affect interactions between the inoculated fungi, particularly if they compete for limited ecological niches. Consequently, the lack of normalization in cell numbers represents a limitation in our study when interpreting inter-*Candida* competition.

Evaluating the region-specific longitudinal colonization of *C. albicans* and *C. parapsilosis* is complex, given that both the proximal and distal colon compartments received luminal suspension from their preceding compartments three times daily. Yet, by the end of the eubiosis period, *C. albicans* and *C. parapsilosis* concentrations were higher in both colon compartments of donor 1 and 2, respectively, as compared to their preceding ileum, inferring active growth. This higher level may stem from the longer retention time in the colon compartments. To further substantiate our observations, we estimated the specific growth rates of each fungal species in each intestine compartment. We direct the reader to the Supplementary Specific growth rate for the complete overview of the methods used and results obtained while only summarizing here our main findings. Higher specific growth rates of *C. albicans* and *C. parapsilosis* in the ileum compartment compared to the colon ones were observed, suggesting that the lower bacterial diversity, and thus colonization resistance, and the presence of more abundant and diverse nutrients favoured their growth. Nonetheless, both fungal species demonstrated active growth in the colon compartments. Further research is, however, required to confirm the growth capacity of each fungal species in separate SHIME^®^ colonic regions.

The production of lactate and the ratio between the three SCFAs remained constant throughout the eubiosis study. This observation suggests a degree of stability in microbiome activity and diversity. Acetate and lactate are known to be converted to propionate and butyrate by various colonic microbes (Martin-Gallausiaux et al. 2021). Hence, the absence of alterations in metabolic profiles suggests a consistent cross-feeding pattern implying that the microbiome composition and its associated metabolic activity did not drastically change. This stability was further confirmed by the observation of a consistent microbiome composition at phylum and genus levels. Moreover, the bacteriome α-diversity remained unaffected, and β-diversity displayed no significant clustering on PCA, DAPC, or RDA throughout the eubiosis period. Similar findings were observed in mice in absence of antibiotic treatment (Gutierrez et al. 2020, McDonough et al. 2021). The fact that artificial inoculation of *Candida* species in the Mucosal Ileum-SHIME^®^ did not disturb the gut microbiome diversity is particularly relevant considering we aimed to simulate *Candida* commensal lifestyle in our *in vitro* model, which should not be associated with bacterial disruptions under eubiosis conditions.

The commensal-to-pathogen transition of *Candida* is often associated with bacterial dysbiosis and *Candida* outgrowth (Zhai et al. 2020). However, the specific bacterial species and their metabolic functions involved in this transition remain poorly understood. Therefore, our second objective was to induce bacterial dysbiosis using clindamycin and study the commensal-to-pathogen transition of *Candida*. Clindamycin is a lincosamide antibiotic known for its broad-spectrum activity against aerobic Gram-positive cocci and a range of anaerobic Gram-positive and Gram-negative bacteria (Klainer 1987). It has previously been shown to induce outgrowth of the mycobiome *in vitro* (Marsaux et al. 2023), and of *C. albicans* specifically in mice (Fan et al. 2015).

While the total bacterial concentration statistically and consistently decreased in the three ileum compartments during the 2 weeks of antibiotic administration, limited impact was observed in the simulated colon microbiomes. The distinction between the ileal and colonic microbiomes lies in bacterial diversity, which was substantially lower in the ileum. Consequently, the ileal microbiome may exhibit limited resilience and resistance to antibiotic treatment, as was previously observed in the SHIME^®^ comparing babies and adults (Marsaux et al. 2023). Despite the limited impact on the colon total bacterial concentrations, bacterial activity, especially butyrate and acetate, was significantly reduced across all intestinal regions. As a result, a substantial shift occurred towards higher propionate production compared to butyrate, which did not reverse upon discontinuation of the antibiotic treatment.

The 16S rRNA gene-targeted sequencing analysis further supported a strong shift at community composition levels due to antibiotic treatment. For instance, there was a drastic shift from *Bifidobacterium* and *Streptococcus* to *Bacteroides* (supporting the increased propionate production), *Enterococcus*, and *Lachnoclostridium* dominance in the colon microbiome of donor 1. These genera remained largely stable, with only *Faecalibacterium* showing substantial recovery once the antibiotic administration ceased, likely contributing to the reincreased butyrate production. Additionally, bacteriome α-diversity decreased during the antibiotic treatment but recovered by the end of the experiment, except in the distal colon, where it remained lower. The β-diversity analysis revealed significant clustering on PCA, DAPC, or RDA, with samples before antibiotic treatment separated from all other samples. Importantly, it is likely that a more long-lasting shift happened in the SHIME^®^ model compared to *in vivo*, because there is no reintroduction of gut-viable microbes from the environment. Nonetheless, similar findings have been reported in humans treated with ciprofloxacin (Dethlefsen and Relman 2011), whereby the microbiome eventually reached a stable but distinct composition 2 months after the treatment, without pathological symptoms associated.

Due to the changes in bacteriome activity and composition, *C. albicans, C. parapsilosis*, and *C. tropicalis*, but not *N. glabratus* and *S. cerevisiae* concentrations, significantly increased during the first week of antibiotic treatment, before their reintroduction. This observation is particularly significant for *C. tropicalis*, as it had fallen below the limit of quantification under eubiosis conditions. However, its resurgence suggests that it was still present in the compartments, albeit at levels too low to be quantifiable. Similar findings in both baby and adult SHIME^®^ studies were previously reported (Marsaux et al. 2023), indicating that opportunistic fungi may be unquantifiable under eubiosis conditions using molecular-based methods. This implies that the prevalence of some *Candida* species may be underestimated in healthy individuals.

Following their reintroduction into the bioreactors, the concentrations of all fungal species increased during the second week of antibiotic treatment. However, *S. cerevisiae* levels rapidly declined, confirming its inability to grow. By contrast, *N. glabratus* concentrations, unquantifiable until its reinoculation, remained stable in the ileum compartments, whereas it varied among donors in the colon compartments. The ability of *N. glabratus* to colonize under conditions of dysbiosis but not eubiosis might be the consequence of a reduced bacteria α-diversity, which provides a reduced colonization resistance towards opportunistic fungi, as recently suggested (Marsaux et al. 2023), and/or opens up an ecological niche. However, the estimated specific growth rates (Supplementary Specific growth rate) showed that *C. tropicalis* and *N. glabratus* did not grow in any colon compartments, whereas *C. parapsilosis* did in all the simulated proximal colons. Additionally, the specific growth rates of *C. albicans* in the simulated proximal colons seemed to inversely correlate with the increased concentrations in the simulated ilea, leading to cell decay during the second week of antibiotic treatment.

Our findings suggest that the source of *Candida* ultimately causing infections might be species-dependent. While *N. glabratus* might originate from a contaminated environment and is only able to colonize vulnerable individuals, like other nosocomial pathogens, *C. albicans* infections may arise from the endogenous microbiome itself, as suggested by other reports (Gabaldon and Fairhead 2019, Zhai et al. 2020). However, as all fungi were inoculated together, it must be noted that we cannot exclude interspecies competition in the different gut compartments, resulting in these different patterns of colonization.

The ability to persist in a recovering microbiome, upon cessation of antibiotic treatment, exhibited species-, intestinal region-, and donor-dependent variations. Concentrations of *C. albicans*, and to a lesser extent *C. tropicalis*, remained stable across all intestine compartments, whereas *N. glabratus* concentrations varied between donors. *Candida parapsilosis* concentrations remained relatively high and stable in the colon compartments compared to the simulated ileum, confirming its adaptation. These novel observations prompt questions about the long-term effects of antibiotics, a well-documented phenomenon for bacteria but less understood in the context of the mycobiome (Dethlefsen and Relman 2011, Seelbinder et al. 2020). Seelbinder et al. (2020) demonstrated that while the bacteriome recovered about 30 days postantibiotic treatment, the mycobiome was shifted from mutualism to competition in humans. Compared to their study, our results underscore the variability in colonic microbiome recovery among individuals, indicating different susceptibilities to long-lasting opportunistic emergence.

Finally, ethanol production mirrored the kinetics of *S. cerevisiae* and *N. glabratus*, with a significant increase occurring only during the second week of antibiotic treatment, eventually returning to pretreatment levels by the end of the experiment. Notably, *S. cerevisiae* is known for its ability to perform alcoholic fermentation from glucose or fructose to ethanol (Maicas 2020). The contribution of the *Candida* species to produce ethanol under limited oxygen conditions remains to be determined. Importantly, the role of ethanol produced by the microbiome, both in general (Meijnikman et al. 2022) and by fungi in particular (Demir et al. 2022), in nonalcoholic fatty liver disease has been proposed. In our study, we observe a correlation between antibiotic use, colonic fungi, and ethanol production, providing a foundation for further research.

## Conclusions

In this study, we investigated the spatial and temporal colonization and growth dynamics of *Candida* species in a multicompartmental semidynamic gastrointestinal system. Our research revealed intriguing insights into how these fungal species interact with the intestinal environment and provided critical information about their behaviour under eubiosis and dysbiosis conditions. First, our findings highlighted the variability in the colonization potential of *Candida* species across different intestinal regions. The ileum compartment proved to be the most favourable environment for *C. albicans* and *C. parapsilosis* under conditions of eubiosis. Second, antibiotic-induced dysbiosis had a significant impact on both the fungal (mycobiome) and bacterial (bacteriome) communities in the gastrointestinal system. This disruption resulted in shifts in bacterial diversity and metabolite production. Notably, opportunistic *Candida* species, especially *C. tropicalis* and *C. albicans*, experienced a resurgence during antibiotic-induced dysbiosis. These species were unquantifiable in some conditions under eubiosis conditions using molecular-based approaches, suggesting that the prevalence of *Candida* presence may be underestimated in healthy individuals. *Nakaseomyces glabratus* grew under conditions of dysbiosis, but not eubiosis. Hence, our study also shed light on the source of *Candida* infections, which may vary depending on the species involved. *Nakaseomyces glabratus*, which colonized dysbiosed microbiome only, might originate from external environments and pose a risk to vulnerable individuals, while *C. albicans* infections could arise from the endogenous microbiome. Interspecies competition and microbiome diversity were identified as potential factors influencing colonization patterns. Understanding the factors driving fungal colonization and the consequence of microbial disruption is crucial for elucidating the dynamics of commensal and pathogenic fungal communities in the gut. Future research should focus on identifying specific bacterial species and their associated microbial functions influencing colonization resistance and explore the long-term effects of antibiotics on the mycobiome and bacteriome.

## Institutional review board

The study was conducted in accordance with the Declaration of Helsinki and approved by the Ethics Committee of the University Hospital Ghent (reference number B670201836585).

## Informed consent

Informed consent of donors was obtained after providing them with detailed information about the project and the use of the samples.

## Supplementary Material

fiae113_Supplemental_Files

## Data Availability

The raw Illumina sequences and corresponding metadata can be consulted under the accession number PRJNA1041351.
