# Supplementary material for: Candida species-specific colonization in the healthy and impaired human gastrointestinal tract as simulated using the Mucosal Ileum-SHIME® model
Source: FEMS Microbiol Ecol. 2024 Aug 21;100(9):fiae113. doi: 10.1093/femsec/fiae113 (PMC11350379; doi:10.1093/femsec/fiae113)
Supplement: fiae113_Supplemental_Files [file fiae113_supplemental_files.zip › Specific growth rate_Supplementary Data_v2_Clean.docx]

***Candida* species-specific colonization in the healthy and impaired human gastrointestinal tract as simulated using the Mucosal Ileum-SHIME® model**

Benoît Marsaux ^a,b,#^, Frédéric Moens ^a^, Gies Vandevijver ^a^, Massimo Marzorati ^a,b^, Tom Van de Wiele ^a,b^

^a^ ProDigest B.V., Ghent, Belgium

^b^ CMET, Ghent University, Ghent, Belgium

**^#^** Correspondence: [Benoit.Marsaux@Prodigest.eu](mailto:Benoit.Marsaux@Prodigest.eu)

SUPPLEMENTARY

Evaluating the specific growth rate of each fungal species in the mucosal Ileum-SHIME® model under conditions of eubiosis and dysbiosis

Evaluating the region-specific longitudinal colonization of each *Candida* and *Saccharomyces* strains inoculated in the mucosal Ileum-SHIME® model is complex, given that both the proximal and distal colon compartments received luminal suspension from their preceding compartments three times daily. However, knowing the volumes of fresh medium entering the ileum, fibre solution entering the proximal colon compartment, and suspension transferred from one preceding compartment to the following one, it is possible to estimate the number of cells produced during each cycle in each compartment separately. From this estimated cell production, it is possible to estimate the specific growth rate. The latter gives insight into the capacity of a given strain to grow under the applied physiological conditions and environmental stress, and will also help deciphering whether each fungal species managed to grow in all the intestine compartments.

**MATERIAL AND METHODS**

An overview of key aspects that will be described below is summarized in Figure S3.

The number of cells produced during each feeding cycle is calculated as follows:

$$\boldsymbol{P=}\frac{\left[ \boldsymbol{Cf} \right]\boldsymbol{-[Ci]}}{\boldsymbol{dt}}$$

Where:

P = number of cells per mL produced during one feeding cycle between time f and time i

dt = considered time frame of cell production (*i.e.,* 1 cycle)

[Cf] = concentration of the fungal species at the end of the cycle (afu/mL)

[Ci] = concentration of the fungal species at the beginning of the cycle (afu/mL)

However, the concentration of each fungal species was only measured every two to three days, with each day containing three feeding cycles. The concentrations at the end of each feeding cycle between two time points of sampling are unknown. These unknown values were estimated by linearly extrapolating the measured values.

$$\left[ \boldsymbol{Cu} \right]\boldsymbol{=}\left[ \boldsymbol{Ct} \right]\boldsymbol{+}\frac{\boldsymbol{u}}{\boldsymbol{n}} \left( \left[ \boldsymbol{CT} \right]\boldsymbol{-[Ct]} \right)$$

Where:

[Cu] = unknown concentration of the fungal species at the end of the feeding cycle u between time points t and T (afu/mL)

[Ct] = measured concentration of the fungal species at the end of the feeding cycle t (afu/mL)

[CT] = measured concentration of the fungal species at the end of the feeding cycle T (afu/mL)

u = number of the feeding cycle

n = total number of feeding cycles between time points t and T

Knowing the concentration of the fungal species at the end of each cycle throughout the entirety of the experiment, it is then possible to estimate the concentration at the beginning of each cycle. This concentration is equal to the one that was measured at the end of the previous cycle though diluted with either fresh medium or fibre solution for the simulated ileum or proximal colon respectively. In addition, the proximal and distal colon compartments received suspension from their preceding intestinal compartment, also potentially containing cells of the species of interest. Importantly, the ileum compartment received fresh feed and experienced a period of incubation (1 h 30 min) before the ileal suspension was transferred to the following colon compartments, whereas the proximal and distal colon compartments received simultaneously a fibre solution (in the proximal colon compartment only), and a luminal suspension from their preceding reactor.

Therefore,

For the ileum:

$$\left[ \boldsymbol{Ci} \right]\boldsymbol{=}\left[ \boldsymbol{C}\boldsymbol{i}^{\boldsymbol{'}}\boldsymbol{,ile} \right]\left( \frac{\boldsymbol{Vr,ile}}{\boldsymbol{Vtot,ile}} \right)$$

Where:

[Ci’,ile] = Concentration of the fungal species in the ileum compartment at the end of the previous feeding cycle (afu/mL)

Vr,ile = Residual volume present in the ileum compartment (Vr,ile = 75 mL)

Vtot,ile = Total volume present in the ileum compartment including the residual volume, and the fresh feed added at the beginning of each feeding cycle (Vtot,ile = 75 + 150 mL)

For the proximal colon:

$$\left[ \boldsymbol{Ci} \right]\boldsymbol{=}\left[ \boldsymbol{C}\boldsymbol{i}^{\boldsymbol{'}}\boldsymbol{,pc} \right]\left( \frac{\boldsymbol{Vr,pc}}{\boldsymbol{Vtot,pc}} \right)\boldsymbol{+}\left[ \boldsymbol{C}\boldsymbol{i}^{\boldsymbol{'}}\boldsymbol{,ile} \right]\left( \frac{\boldsymbol{Vr,ile}}{\boldsymbol{Vtot,ile}} \right)\left( \frac{\boldsymbol{Vile\to pc}}{\boldsymbol{Vtot,pc}} \right)$$

Where:

[Ci’,pc] = Concentration of the fungal species in the proximal colon compartment at the end of the previous feeding cycle (afu/mL)

Vr,pc = Residual volume present in the proximal colon compartment (Vr,pc = 500 mL)

Vtot,pc = Total volume present in the proximal colon compartment including the residual volume, the fresh fibre solution added at the beginning of each feeding cycle, and the volume of ileal suspension transferred into the proximal colon compartment at the beginning of each feeding cycle (Vtot,pc = 500 + 50 + 150 mL)

Vile$\to$pc = Volume of ileal suspension transferred into the proximal colon compartment at the beginning of each feeding cycle (Vile$\to$pc = 150 mL)

For the distal colon:

$$\left[ \boldsymbol{Ci} \right]\boldsymbol{=}\left[ \boldsymbol{C}\boldsymbol{i}^{\boldsymbol{'}}\boldsymbol{,dc} \right]\left( \frac{\boldsymbol{Vr,dc}}{\boldsymbol{Vtot,dc}} \right)\boldsymbol{+}\left[ \boldsymbol{C}\boldsymbol{i}^{\boldsymbol{'}}\boldsymbol{,pc} \right]\left( \frac{\boldsymbol{Vpc\to dc}}{\boldsymbol{Vtot,dc}} \right)\boldsymbol{+}\left[ \boldsymbol{C}\boldsymbol{i}^{\boldsymbol{'}}\boldsymbol{,ile} \right]\left( \frac{\boldsymbol{Vr,ile}}{\boldsymbol{Vtot,ile}} \right)\left( \frac{\boldsymbol{Vile\to pc}}{\boldsymbol{Vtot,pc}} \right)\left( \frac{\boldsymbol{Vpc\to dc}}{\boldsymbol{Vtot,dc}} \right)$$

Where:

[Ci’,dc] = Concentration of the fungal species in the distal colon compartment at the end of the previous feeding cycle (afu/mL)

Vr,dc = Residual volume present in the distal colon compartment (Vr,dc = 800 mL)

Vtot,dc = Total volume present in the distal colon compartment including the residual volume, and the volume of proximal suspension transferred into the distal colon compartment at the beginning of each feeding cycle (Vtot,dc = 500 + 200 mL)

Vpc$\to$dc = Volume of proximal colon suspension transferred into the distal colon compartment at the beginning of each feeding cycle (Vpc$\to$dc = 200 mL)

Knowing the concentrations of the fungal species in all intestine compartments both at the beginning and at the end of each cycle enabled to calculate the number of cells produced during each cycle, and to then calculate the specific growth rate for each cycle.

$$\boldsymbol{\mu=}\frac{\boldsymbol{P}}{\boldsymbol{[Ci]}}\boldsymbol{=}\frac{\left[ \boldsymbol{Cf} \right]\boldsymbol{-[Ci]}}{\left[ \boldsymbol{Ci} \right]\boldsymbol{\times dt}}$$

Where:

μ = Specific growth rate (expressed in cycle^-1^, alternatively in hours^-1^, considering 1 cycle = 8 hours)

These calculations are only estimates based on measurements, hence, several limitations need to be considered, and include: (i) concentrations that did not reach the limit of quantification through qPCR were considered zero; (ii) we assumed that no growth happened in the ileal compartment during the incubation period with fresh feed (we considered that microbes remained in stationary phase for an extended period during the previous cycle and hence experienced lag phase); (iii) we assumed that the suspensions were instantly transferred from the ileum to the proximal colon, and from the proximal colon to the distal colon, whereas it took c.a., 30 and 40 min, respectively; (iv) we linearly extrapolated the concentrations between two sampling points while no information was available to confirm this linear behaviour.

**RESULTS**

## **Specific growth rates of the four Candida species and Saccharomyces cerevisiae in eubiosis conditions**

The specific growth rates of the five inoculated fungal species were estimated across all intestinal compartments during both the eubiosis and the dysbiosis studies, and the recovery period.

In eubiosis conditions, *C. albicans* and *C. parapsilosis* displayed relatively constant and similar growth rates in all ileum compartments (Figure S4, Figure S5). Any variations in the specific growth rates of these two species were likely attributed to calculation biases. *Candida tropicalis* exhibited inconsistent specific growth rates, occasionally reaching maximum values similar to those of *C. albicans* and *C. parapsilosis* (ca., 0.2 – 0.3 h^-1^). After their inoculation, the concentrations of *N. glabratus* and *S. cerevisiae* rapidly decreased under the limit of quantification resulting in a zero value for the calculated growth rate throughout the remainder of the eubiosis study.

On the contrary, in the colon compartments, cell concentrations of *C. albicans* and *C. parapsilosis* declined during the first two weeks post-inoculation which translated into negative values for the estimated growth rate. They showed intermediate growth, albeit at lower specific growth rates compared to the ileum compartments, during the third week of the eubiosis study, particularly in the proximal colon compartments*.* *Candida tropicalis*, *N. glabratus*, and *S. cerevisiae* cell concentrations, on the other hand, consistently decreased throughout the entire eubiosis study in all colon compartments resulting in concentrations below the limit of quantification and thus a specific growth rate value of zero.

## **Specific growth rates of the four Candida species and Saccharomyces cerevisiae in dysbiosis conditions**

Dosing the ileal compartments with clindamycin did not appear to significantly impact the specific growth rates of *C. albicans* and *C. parapsilosis* (Figure S4, Figure S5). In contrast, the specific growth rates values of *C. tropicalis* were mostly positive (ca., 0.3 h^-1^) throughout the first week of antibiotic treatment, confirming its active growth. Therefore, this species was not completely washed-out of the ileal compartment during the eubiosis study; it initiated opportunistic growth in the presence of a dysbiosed bacterial community. During this period, neither *N. glabratus* or *S. cerevisiae* exhibited growth. The re-inoculation of the five fungal species did not alter the specific growth rates of *C. albicans* and *C. tropicalis*, whereas the rates decreased for *C. parapsilosis* to ca., 0 – 0.2 h^-1^. Interestingly, despite the fact that *N. glabratus* grew in the ileal compartments , its specific growth rates consistently decreased. On the contrary*, S. cerevisiae* mostly experienced cell death during the second week of antibiotic treatment. Similar patterns were observed during the recovery period, except for *C. albicans*, for which the specific growth rates tended to decrease by the end of the experiment.

In contrast to the consistent behaviours among the three ileal reactors for the five fungal species, some donor-specific differences were observed in the proximal colon compartment upon dosing the bioreactors with the antibiotic treatment. While the specific growth rates of *C. albicans* remained constant and positive in the bioreactors inoculated with the faecal samples from adult donors 1 and 2, cells decayed in the bioreactor from adult donor 3. Furthermore, no growth of *N. glabratus* was observed in the bioreactors from adult donors 1 and 2, whereas positive specific growth rates were observed in the bioreactor from adult donor 3. However, similar trends were observed among all proximal colon compartments regarding the specific growth rates of *C. parapsilosis*, which increased upon dosing with antibiotics, *C. tropicalis*, for which cells mainly decayed, and *S. cerevisiae*, which showed no growth. Cells consistently decayed upon the re-introduction of the fungal species during the second week of antibiotic treatment and the recovery period in all proximal colon compartments, except for *C. parapsilosis.* For this species, the specific growth rates initially decreased but returned to initial values by the end of the antibiotic period, and remained unaltered throughout the recovery period.

In contrast to the proximal colon compartments, similar patterns were observed across all distal colon compartments. Cells consistently decayed throughout the dysbiosis and recovery periods, regardless of the re-inoculation of the fungal species in the bioreactors.

**DISCUSSION**

In summary, our observations underscored that the ileum compartment supported a more optimal growth environment for *C. albicans* and *C. parapsilosis* under conditions of eubiosis, as evidenced through their higher specific growth rates in this intestinal region compared to the colonic ones. This favourable environment may be attributed to factors such as lower bacterial cell density and/or diversity, resulting in reduced competition or antagonism, and the presence of more abundant and diverse nutrients. Compared to the reported maximum specific growth rates (1.5-4.9 h^-1^) found in literature and under pathogenic conditions (1) our calculated values seem quite plausible given the fact that we establish higher levels of colonization resistance in the M-SHIME® system. The anaerobic and glucose-poor environment contributes also to lowering the specific growth rates of the fungal species in our *in vitro* model.

Interestingly*,* calculating the specific growth rates of *C. albicans* and *C. parapsilosis* demonstrated their active growth, albeit partially, in both the proximal and distal colon compartments, following an initial two-week period characterized primarily by cell decay. In contrast, *N. glabratus* and *S. cerevisiae* appear unable to grow in any of the intestine compartments, confirming previous observations that these species do not seem to be adapted to thrive under eubiotic conditions.

Remodelling the microbial diversity, but not the total bacterial concentrations, reduced the colonization resistance towards *C. tropicalis* and *N. glabratus,* enabling their growth under conditions of dysbiosis in all the ileal compartments. Indeed, the cell concentrations and specific growth rates of the two fungal species increased. However, this was not observed in the colon compartments, suggesting the maintenance of colonization resistance during antibiotic treatment*.*

**REFERENCE**

1. Pang SM, Tristram S, Brown S. 2010. The Contribution of Growth Rate to the Pathogenicity of Candida spp.. International Journal of Medicine and Medical Sciences 1:2.
