# Supplementary material for: Candida species-specific colonization in the healthy and impaired human gastrointestinal tract as simulated using the Mucosal Ileum-SHIME® model
Source: FEMS Microbiol Ecol. 2024 Aug 21;100(9):fiae113. doi: 10.1093/femsec/fiae113 (PMC11350379; doi:10.1093/femsec/fiae113)
Supplement: fiae113_Supplemental_Files [file fiae113_supplemental_files.zip › Supplementary data_Figure_Legends_v1.docx]

**Supplementary Figure Legends**

**Figure S1.** *Candida albicans* (A), *C. parapsilosis* (B), *C. tropicalis* (C), *S. cerevisiae* (D), *N. glabratus* (E), and total bacteria concentrations (F) measured by qPCR and total live bacteria concentrations measured by flow cytometry (G) (box and whiskers showing minimum and maximum values) in the ileum (black), and the proximal (brown) and distal (purple) colon compartments inoculated at day 0 with the faecal samples from donor 1 (green), 2 (red) or 3 (blue). The fungal species values were corrected with flow cytometry data obtained on their respective pure overnight culture, hence resulting in cell counts instead of gene copy counts. During the eubiosis study, the concentrations were compared for change between the baseline period (average of d19 – d35), the week 1 of the eubiosis period (d36 – d42), and the weeks 2 and 3 of the eubiosis period (d44 – d56). During the dysbiosis study, the concentrations were compared for change between the week 3 of the eubiosis period (d51 – d56), the first week of the dysbiosis period (d58 to d63), the second week of the dysbiosis period (d64 – d70), and the recovery period (d71 – d79). Significant differences were assessed using Kruskal-Wallis tests with Dunn’s multiple comparisons, and are marked with asterisks (* p < 0.05, ** p < 0.01, *** p < 0.001, **** p < 0.0001). AB = antibiotic; LOQ = Limit of quantification.

**Figure S2.** Lactate (A), acetate (B), ethanol (C), propionate (D), and butyrate (E) concentrations (box and whiskers showing minimum and maximum values) in the ileum (black), and the proximal (brown) and distal (purple) colon compartments inoculated at day 0 with the faecal samples from donor 1 (green), 2 (red) or 3 (blue). During the eubiosis study, the concentrations were compared for change between the baseline period (average of d19 – d35), the week 1 of the eubiosis period (d36 – d42), and the weeks 2 and 3 of the eubiosis period (d44 – d56). During the dysbiosis study, the concentrations were compared for change between the week 3 of the eubiosis period (d51 – d56), the first week of the dysbiosis period (d58 – d63), the second week of the dysbiosis period (d64 – d70), and the recovery period (d71 – d79). Significant differences were assessed using Kruskal-Wallis tests with Dunn’s multiple comparisons, and are marked with asterisks (* p < 0.05, ** p < 0.01, *** p < 0.001, **** p < 0.0001). AB = antibiotic

**Figure S3.** Schematic representation of the Mucosal Ileum-SHIME® *in vitro* model (A), and of the timeline consisting of n feeding cycles in between two sampling points t and T (B). (A) The ileum compartment is characterized by a residual volume of 75 mL and receives three times daily 150 mL of fresh feed. The proximal colon has a residual volume of 500 mL and receives three times daily 50 mL of fresh fiber solution and 150 mL of ileal suspension. The distal colon has a residual volume of 800 mL and receives three times daily 200 mL of proximal colon suspension. (B) Between two sampling time points t and T, n feeding cycles happened. The fungal species of interest reaches a concentration [Ci’] or [Cf] by the end of a cycle, while having a concentration of [Ci] at the start of the cycle. The concentration [Ci] can be estimated from the concentration [Ci’] by taking into considerations the dilution effects from fresh feed, fresh fiber solution, or the suspension coming from the preceding intestinal compartment, the latter potentially also containing the species of interest. The number of cells P produced during one cycle is equal to the difference between the start and end concentrations of the given cycle u. Finally, each unknown concentration [Cu] between two sampling points were linearly extrapolated. PC = proximal colon; DC = distal colon.

**Figure S4.** *Candida albicans*, *C. parapsilosis*, *C. tropicalis*, *N. glabratus*, and *S. cerevisiae* calculated specific growth rates at each measured time point in the ileum, proximal, and distal colon compartments. The colon compartments were inoculated at day 0 with the faecal samples from donor 1 (green), 2 (red) or 3 (blue). All intestine compartments were inoculated with the five fungal species (red line) at days 35 to 37 during the eubiosis study, and at days 63 to 65 during the dysbiosis study. The latter study consisted of dosing the bioreactors three times daily with clindamycin for two weeks (from day 56 to 70). AB = antibiotic; AB+F = antibiotic with fungal re-inoculation.

**Figure S5.** *Candida albicans*, *C. parapsilosis*, *C. tropicalis*, *S. cerevisiae*, and *N. glabratus* calculated specific growth rates (box and whiskers showing minimum and maximum values) in the ileum (black), and the proximal (brown) and distal (purple) colon compartments inoculated at day 0 with the faecal samples from donor 1 (green), 2 (red) or 3 (blue). Values were grouped per week. The days during which the fungal species were inoculated were omitted, as they strongly biased the calculation of the specific growth rates. W = week; AB = Antibiotic; Eub = eubiosis.
