# Supplementary figures and images for: Candida species-specific colonization in the healthy and impaired human gastrointestinal tract as simulated using the Mucosal Ileum-SHIME® model
Source: FEMS Microbiol Ecol. 2024 Aug 21;100(9):fiae113. doi: 10.1093/femsec/fiae113 (PMC11350379; doi:10.1093/femsec/fiae113)

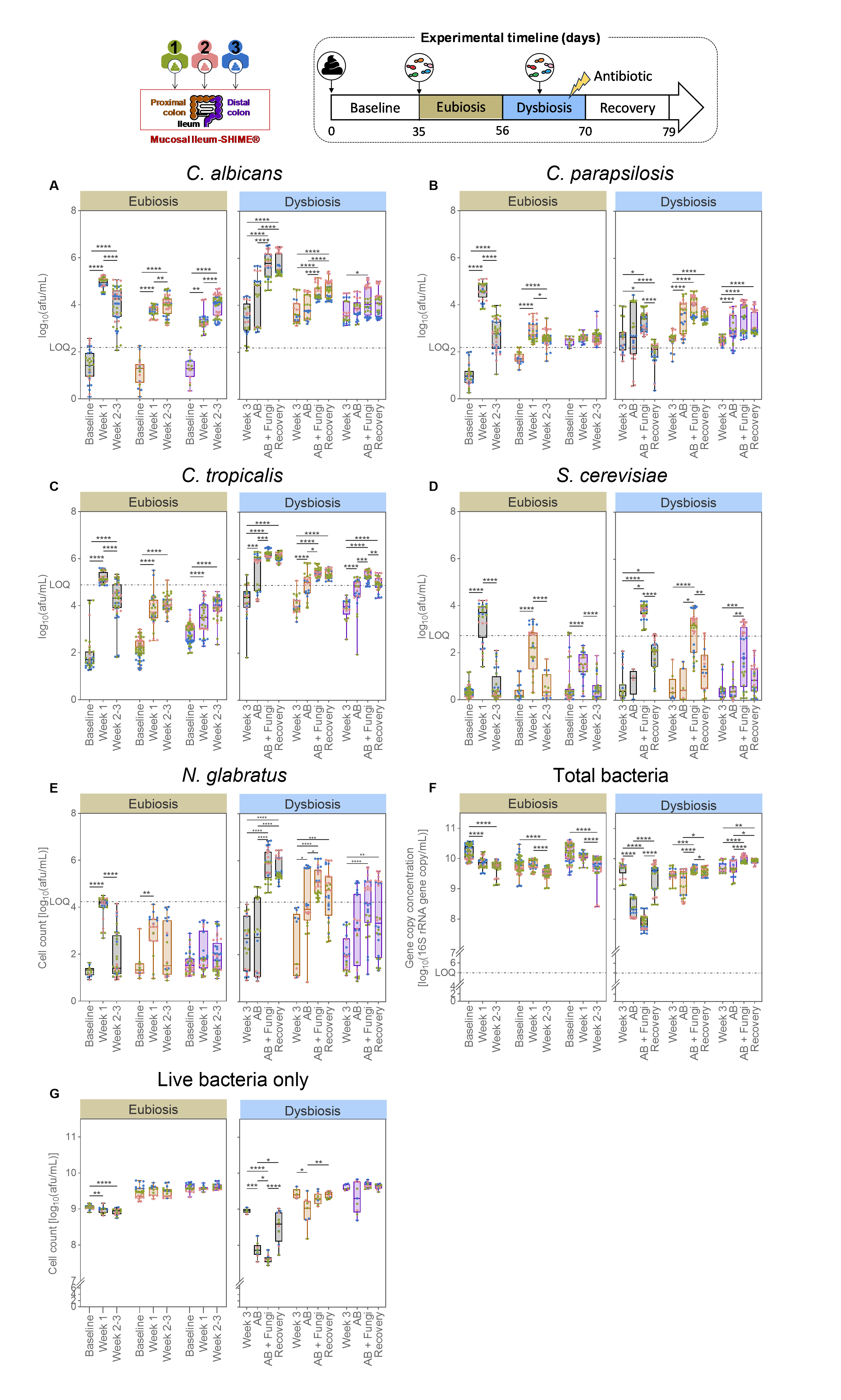

Supplement: fiae113_Supplemental_Files [file fiae113_supplemental_files.zip › Figure S1 - Revised_Supplementary Data - pix.tif]

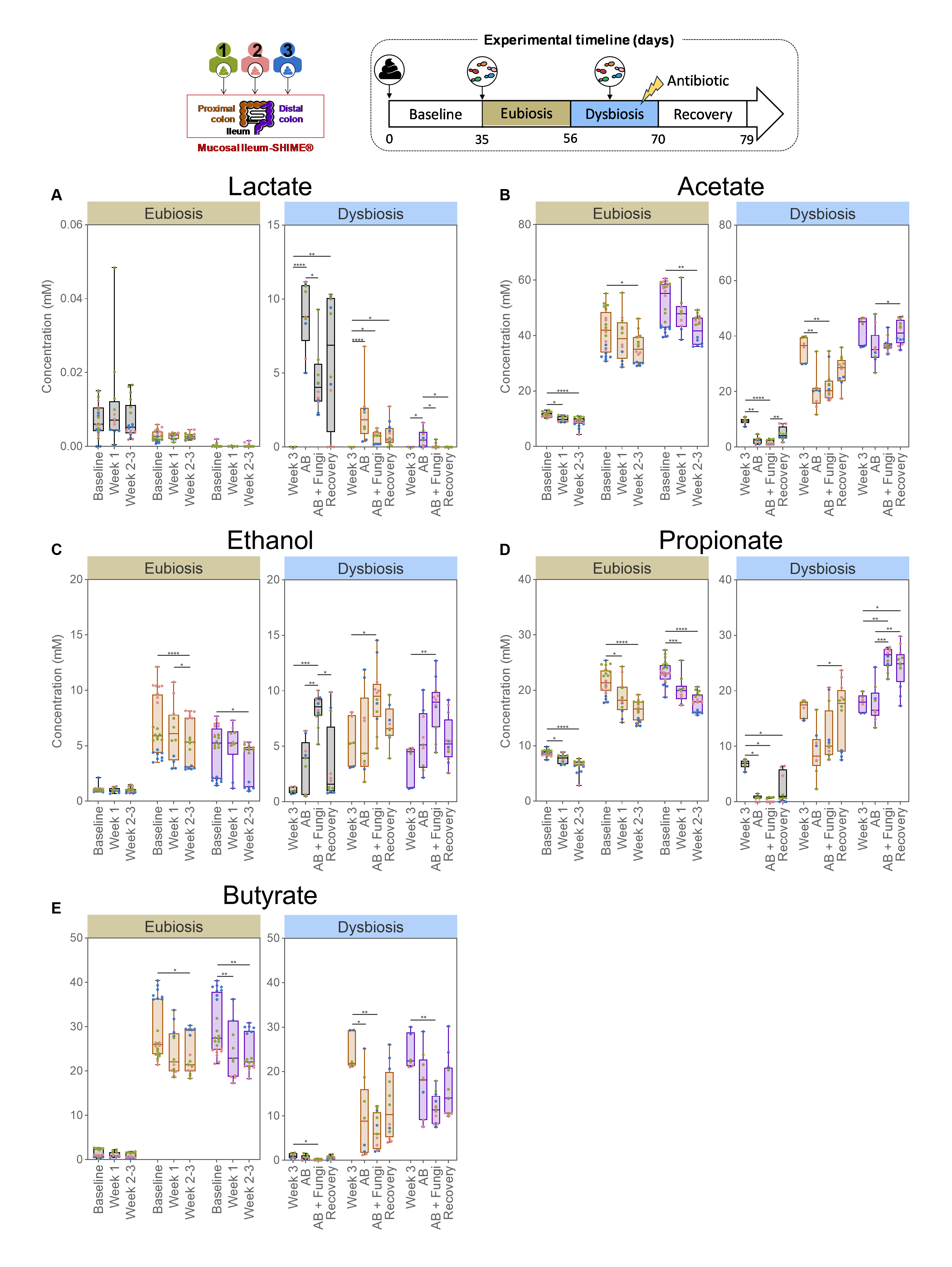

Supplement: fiae113_Supplemental_Files [file fiae113_supplemental_files.zip › Figure S2 - Revised_Supplementary Data - pix.tif]

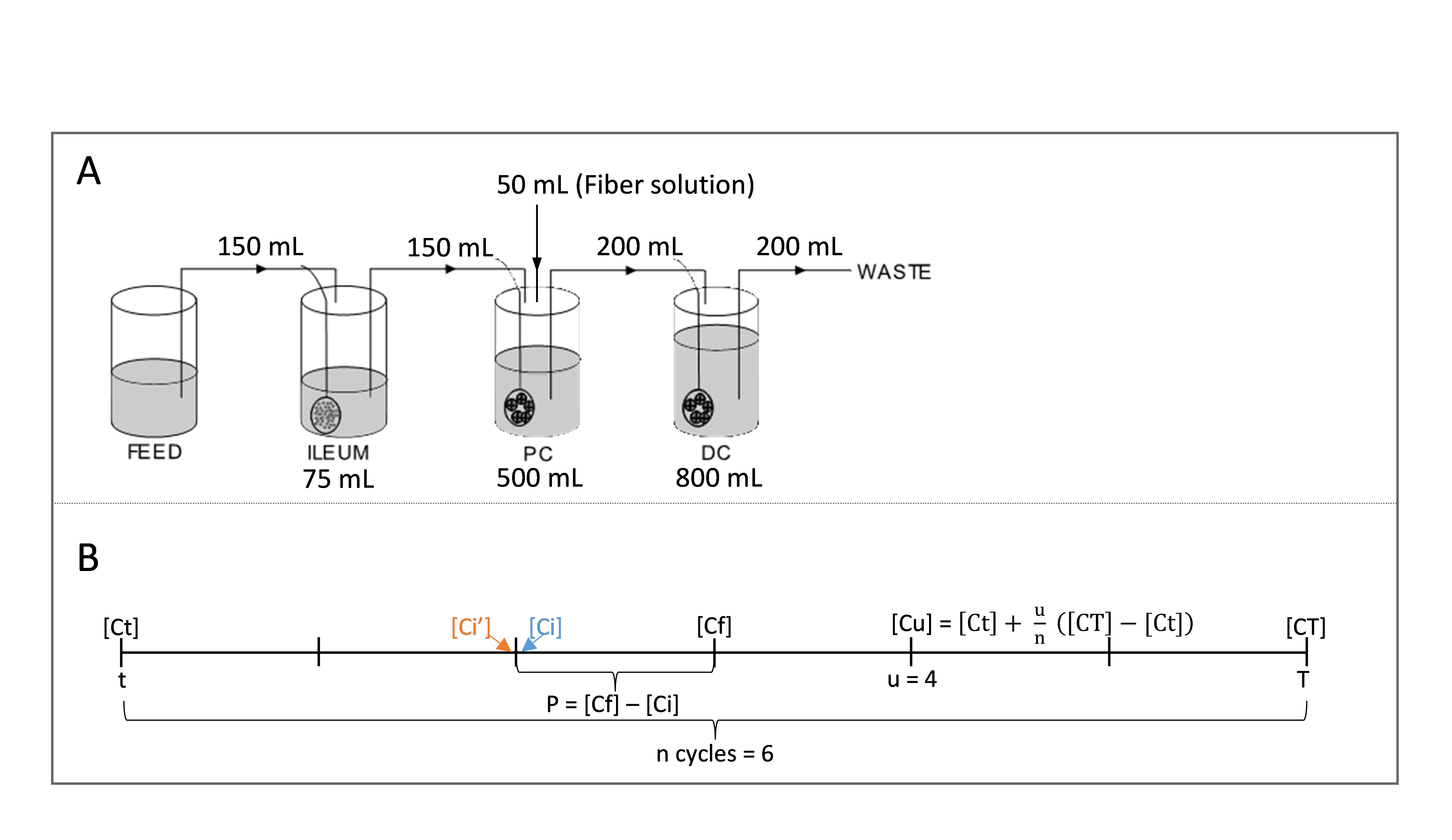

Supplement: fiae113_Supplemental_Files [file fiae113_supplemental_files.zip › Figure S3 - Revised_Supplementary Data_v2.tif]

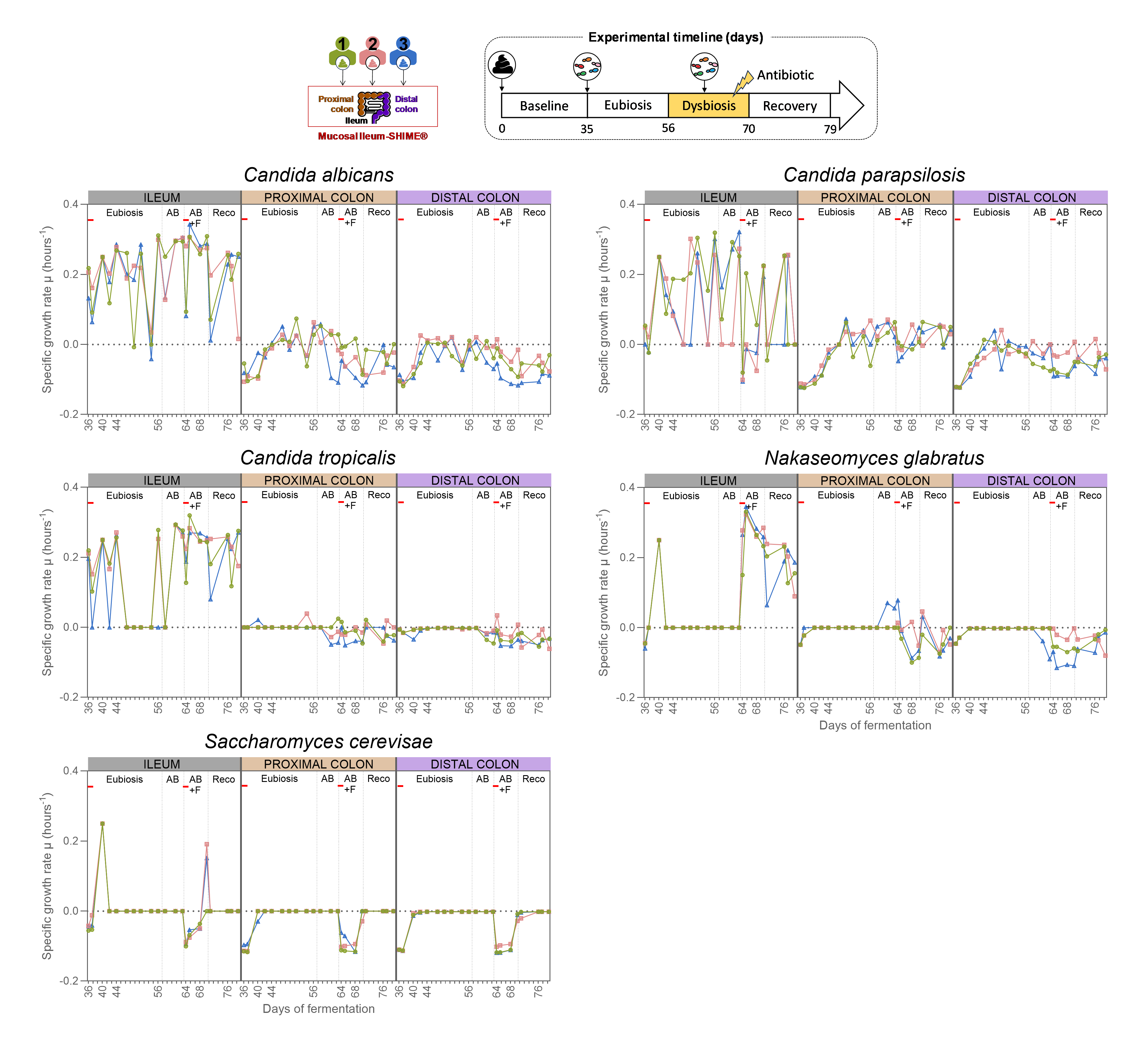

Supplement: fiae113_Supplemental_Files [file fiae113_supplemental_files.zip › Figure S4 - New_Supplementary Data - pix.tif]

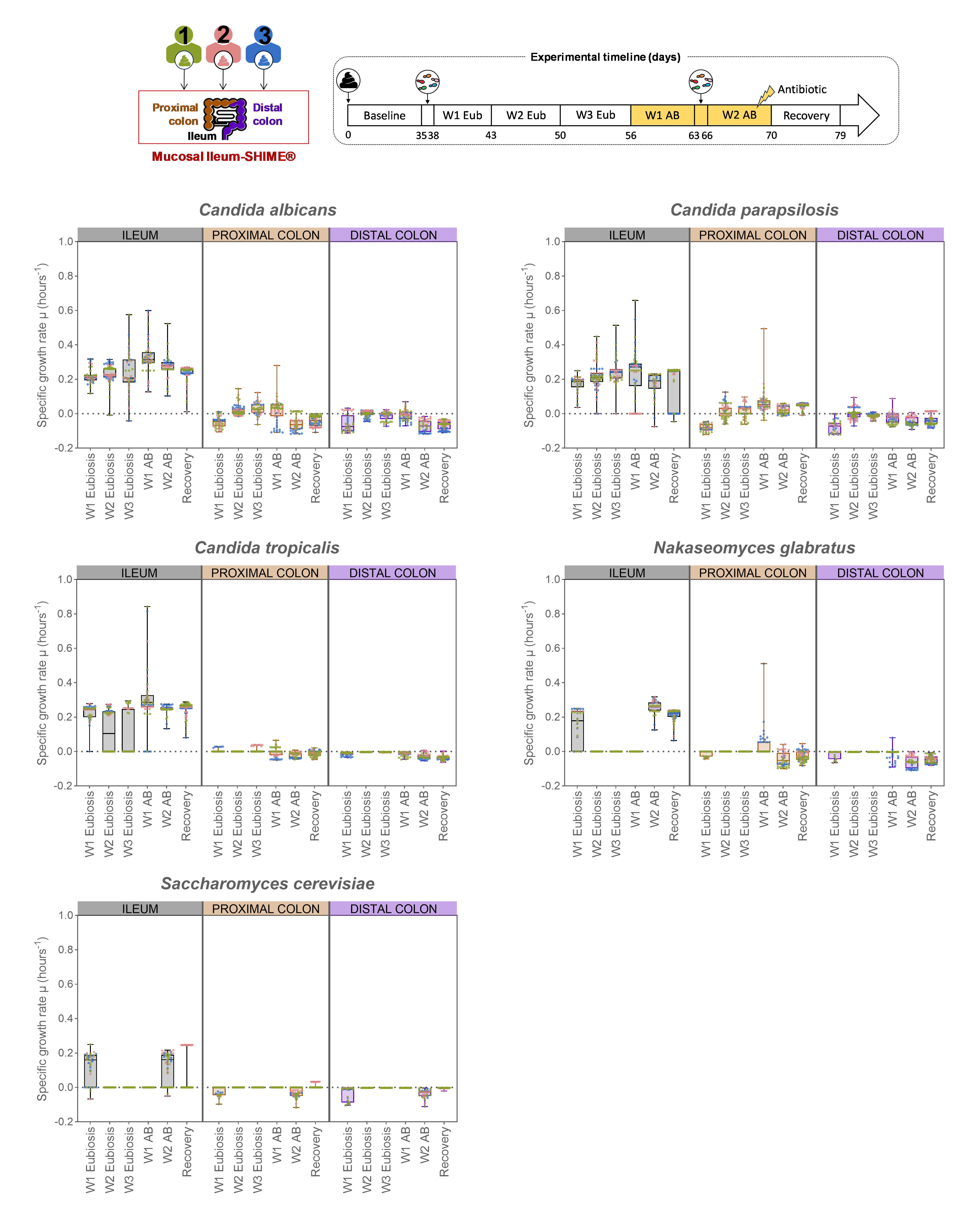

Supplement: fiae113_Supplemental_Files [file fiae113_supplemental_files.zip › Figure S5 - New_Supplementary Data - pix.tif]
